# Translational control of depression-like behavior via phosphorylation of eukaryotic translation initiation factor 4E

**DOI:** 10.1038/s41467-018-04883-5

**Published:** 2018-06-25

**Authors:** Argel Aguilar-Valles, Nabila Haji, Danilo De Gregorio, Edna Matta-Camacho, Mohammad J. Eslamizade, Jelena Popic, Vijendra Sharma, Ruifeng Cao, Christoph Rummel, Arnaud Tanti, Shane Wiebe, Nicolas Nuñez, Stefano Comai, Robert Nadon, Giamal Luheshi, Naguib Mechawar, Gustavo Turecki, Jean-Claude Lacaille, Gabriella Gobbi, Nahum Sonenberg

**Affiliations:** 1 Department of Biochemistry and Goodman Cancer Centre, McGill University, Montreal, QC H3A 1A3 Canada; 2 Department of Neurosciences and Groupe de Recherche sur le Système Nerveux Central (GRSNC), Université de Montréal, Montreal, QC H3A 1A1 Canada; 3 Department of Psychiatry, McGill University, Montreal, QC H3C 3J7 Canada; 4 Institute of Veterinary Physiology and Biochemistry, Justus-Liebig-University Giessen, Giessen, D-35392 Germany; 5 Douglas Mental Health University Institute, McGill University, Montreal, QC H4H 1R3 Canada; 6 Department of Human Genetics, McGill University and Genome Quebec Innovation Centre, Montreal, QC H3A 0G1 Canada; 7 Present Address: Department of Biomedical Sciences, University of Minnesota Medical School, Duluth, MN 55812 USA; 8 Present Address: Faculty of Medicine, Vita-Salute San Raffaele University, Milan, 20132 Italy

## Abstract

Translation of mRNA into protein has a fundamental role in neurodevelopment, plasticity, and memory formation; however, its contribution in the pathophysiology of depressive disorders is not fully understood. We investigated the involvement of MNK1/2 (MAPK-interacting serine/threonine-protein kinase 1 and 2) and their target, eIF4E (eukaryotic initiation factor 4E), in depression-like behavior in mice. Mice carrying a mutation in eIF4E for the MNK1/2 phosphorylation site (Ser209Ala, *Eif4e* ki/ki), the *Mnk1/2* double knockout mice (*Mnk1/2*^−/−^), or mice treated with the MNK1/2 inhibitor, cercosporamide, displayed anxiety- and depression-like behaviors, impaired serotonin-induced excitatory synaptic activity in the prefrontal cortex, and diminished firing of the dorsal raphe neurons. In *Eif4e* ki/ki mice, brain IκBα, was decreased, while the NF-κB target, TNFα was elevated. TNFα inhibition in *Eif4e* ki/ki mice rescued, whereas TNFα administration to wild-type mice mimicked the depression-like behaviors and 5-HT synaptic deficits. We conclude that eIF4E phosphorylation modulates depression-like behavior through regulation of inflammatory responses.

## Abstract (web summary)

Translation of mRNA contributes to neuronal function and complex behaviours, and inflammation is thought to contribute to depression. Here the authors show that mice lacking phosphorylation sites in eIF4E (eukaryotic initiation factor 4E) display anxiety- and depression-like behaviour and decreased IkBα expression; furthermore TNFα delivery to the medial prefrontal cortex induces depression-like behaviour and deficits in serotonergic transmission.

## Introduction

Major depressive disorder (MDD) is one of the leading causes of disability worldwide^1^, with a lifetime prevalence of 16.6 % in the United States^2^. Current treatments are ineffective in about one third of patients^3^, indicating the urgent need to better understand MDD pathophysiology and identify novel therapeutic targets. The MAPK/ERK pathway is impaired in the brains of MDD patients^4,5^, however a causal role for downstream mRNA translation in depression has not been studied. mRNA translation (protein synthesis) plays a major role in the control of gene expression, allowing for rapid and spatially restricted changes in protein levels^6^. A rate-limiting step in the initiation of mRNA translation is the recruitment of the eukaryotic initiation factor 4E (eIF4E) to the mRNA 5′cap structure (m7GpppN, where N is any nucleotide). eIF4E binds to the scaffolding protein eIF4G, which also binds to the RNA helicase eIF4A, to form the eIF4F complex, which mediates the recruitment of preinitiation complex^6^. eIF4E is a target of the mammalian target of rapamycin complex 1 (mTORC1) and MAPK/ERK pathways;^7^ the activation of which stimulates protein synthesis.

The MAP kinases ERK1/2 and p38 control mRNA translation through the MAPK-interacting serine/threonine-protein kinase 1 and 2 (MNK1/2). MNKs phosphorylate eIF4E^8^ on Ser209^9–11^. Phosphorylation of eIF4E engenders increased translation of a subset of mRNAs^12^, some of which play a role in memory formation^13^ and regulation of circadian rhythms^14^.

In MDD-suicide subjects, the activity, protein, and mRNA levels of ERK1/2 are decreased in post-mortem prefrontal and hippocampal areas^4,5^. The consequence of reduced activation of ERK1/2 in the pathophysiology of depression and on the anti-depressant effect of selective serotonin reuptake inhibitors (SSRIs)^15,16^ and mood stabilizers^17^ is well understood. In this regard, MAPK kinase (MEK) inhibition induces depression-like behaviors and blocks the antidepressant action of SSRIs and tricyclic compounds^15^. Furthermore, reduction in P38 MAPK phosphorylation has been observed in an animal model of depression and aggressive behavior, induced by dietary deprivation of n-3 polyunsaturated fatty acids^18^. Also, P38 MAPK activation contributes to the induction of depression-like behavior by chronic stress and immune mediators^19,20^.

Here we demonstrate that genetic and pharmacological inhibition of eIF4E phosphorylation in mice resulted in a depression-like state, impaired serotonin excitatory activity in the prefrontal cortex, and diminished firing of raphe serotonergic neurons. Inhibition of eIF4E phosphorylation caused decreased translation of IκBα (nuclear factor of kappa light polypeptide gene enhancer in B-cells inhibitor, alpha) mRNA in the brain. Decreased IκBα, a repressor of the transcription factor NF-κB, led to increased expression of the cytokine TNFα and exacerbated microglial responsiveness to inflammatory stimuli with lipopolysaccharide (LPS). Increased inflammation in the brain is a hallmark of several psychiatric disorders, including MDD^19,21^, in which patients have elevated cytokine levels^22,23^, and microgliosis^24^. Increased peripheral and central inflammatory markers in MDD are thought to contribute to the pathophysiology and treatment responsiveness^19,21^. Importantly, inhibition of TNFα ameliorated the depression-like behavior, as well as serotonergic synaptic and cell firing alterations induced by the lack of eIF4E phosphorylation.

## Results

### The MNK1/2-eIF4E pathway controls depression-like behaviors

**Fig. 1:**
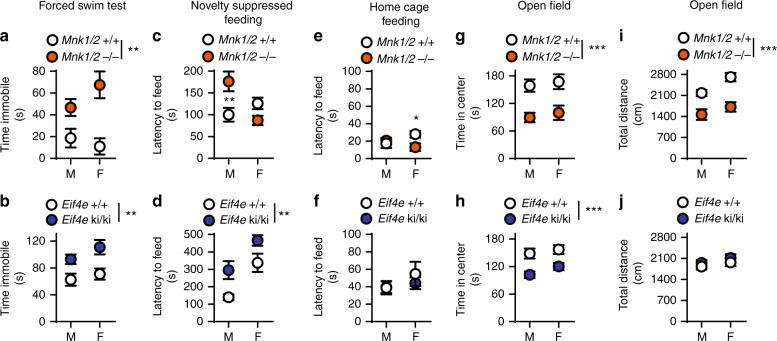
Genetic inhibition of eIF4E phosphorylation induced anxiety- and depression-like behaviors. **a** Male (M) and female (F) mice mutant for the genes encoding MNK1 and MNK2 (*Mnk1/2*^−/−^ mice) and their wild-type littermates (*Mnk1/2*^+/+^) were assessed for despair-like behavior in the forced swim test (FST) (M, *n* = 8 *Mnk1/2*^+/+^, *n* = 9 *Mnk1/2*^−/−^; F, *n* = 3 *Mnk1/2*^+/+^, *n* = 9 *Mnk1/2*^−/−^). **b** Male and female mice bearing a mutation in the *Eif4e* gene (Ser209 was substituted to Ala, *Eif4e* ki/ki) were also tested in the FST (M, *n* = 19 *Eif4e*^+/+^, *n* = 19 *Eif4e* ki/ki; F, *n* = 7 *Eif4e+/+*, *n* = 8 *Eif4e* ki/ki). **c** Novelty suppressed feeding (NSF) was assessed in *Mnk1*/2^−/−^ (M, *n* = 9; F, *n* = 10) and wild-type mice (M, *n* = 8; F, *n* = 10). **d** NSF was also assayed in *Eif4e*^+/+^ and ki/ki mice (M, *n* = 9 *Eif4e*^+/+^, *n* = 9 *Eif4e* ki/ki; F, *n* = 8 *Eif4e*^+/+^, *n* = 9 *Eif4e* ki/ki). **e** Latency to feed in the home cage (home cage feeding, HCF) was determined in the *Mnk1/2*^−/−^ and **f** *Eif4e* ki/ki mice and their littermates. **g** Time spent in the center of an open field (OF) arena was measured in *Mnk1/2*^−/−^ (M, *n* = 12 *Mnk1/2*^+/+^, *n* = 8 *Mnk1/2*^−/−^; F, *n* = 7 *Mnk1/2*^+/+^, *n* = 13 *Mnk1/2*^−/−^) and **h** *Eif4e* ki/ki mice and their littermates (M, *n* = 17 *Eif4e*^+/+^, *n* = 18 *Eif4e* ki/ki; F, *n* = 11 *Eif4e*^+/+^, *n* = 19 *Eif4e* ki/ki). **i** Locomotion was also measured in *Mnk1/2*^−/−^ and **j** *Eif4e* ki/ki mice. ** *p* < 0.01, *** *p* < 0.001 vs. wild-type littermates (see Supplementary Table 2 for detailed results of statistical tests)

To study the role of eIF4E phosphorylation in depression pathophysiology, we examined the *Mnk1* and *Mnk2* double knock-out (*Mnk1/2*^−/−^)^11^, and the *Eif4e* Ser209Ala knock-in (*Eif4e* ki/ki)^12^ mice in several validated paradigms for depression-like behavior. In the forced swim test (FST), immobility time was increased in male (M) and female (F) *Mnk1/2*^−/−^ (by 28.1 ± 13.6 s in M and 56.5 ± 18.7 s in F mice) and *Eif4e* ki/ki mice (by 30.4 ± 10.9 s in M and 40.0 ± 17.3 s in F) (Fig. 1a, b). Male *Mnk1/2*^−/−^ and *Eif4e* ki/ki mice were more immobile than wild-type in the tail suspension test (by 70.2 ± 27.5 s and 35.8 ± 12.0 s, respectively) (Supplementary Fig. 1a, b). This test could not be accurately assessed in females, since a large proportion climbed on their own tails while suspended, as previously reported^25^.

In the novelty-suppressed feeding (NSF) test, a measure of hyponeophagia that is responsive to chronic treatment with antidepressants^26,27^, the latency to feed in a novel context was increased in male *Mnk1/2*^−/−^ (by 73.3 ± 23.4 s) and *Eif4e* ki/ki (by 156.1 ± 56.7 s), as well as female *Eif4e* ki/ki mice as compared to control littermates (by 128.5 ± 57.4 s) (Fig. 1c, d). The mutant mice did not differ in the latency to feed in their home cage as compared to wild-type (HCF, Fig. 1e, f). In contrast, the female *Mnk1/2*^−/−^ were not affected in the latency to feed in the novel context (Fig. 1c) but exhibited a decreased latency to feed in the home cage of 14.8 ± 5.7 s (Fig. 1e). This suggests a female-specific role in feeding behavior for MNK1/2, independent of eIF4E phosphorylation.

Next, we tested exploratory behavior in the open field. Both male and female *Mnk1/2*^−/−^ and *Eif4e* ki/ki mice showed a decrease in the time spent in the center of the arena (*Mnk1/2*^−/−^: by 69.6 ± 21.4 s in M and 67.7 ± 22.0 s in F; *Eif4e* ki/ki: by 46.4 ± 12.1 s in M and 36.6 ± 13.6 s in F) (Fig. 1g, h). While no changes in locomotion were observed in *Eif4e* ki/ki mice compared to their control littermates (Fig. 1j), the *Mnk1/2* mice showed decreased locomotion (by 717.8 ± 214 s in M and 990 ± 219.8 s in F; Fig. 1i). A second exposure to the open field abrogated the difference in locomotion between *Mnk1/2*^−/−^ and wild-type mice (Supplementary Fig. 1c–e). However, the difference in time spent in the center of the field persisted (Supplementary Fig. 1d–f), suggesting that novelty-related anxiety, rather than hypo-activity, caused decreased locomotion during the 1^st^ exposure to the open field in the *Mnk1/2*^−/−^ mice. Taken together, these results demonstrate that reduction of eIF4E phosphorylation, either by knocking out the *Mnk1* and *Mnk2* genes or by mutating the phosphorylation site in *Eif4e*, elicits depression- and anxiety-like behaviors.

### eIF4E phosphorylation controls 5-HT neurotransmission

**Fig. 2:**
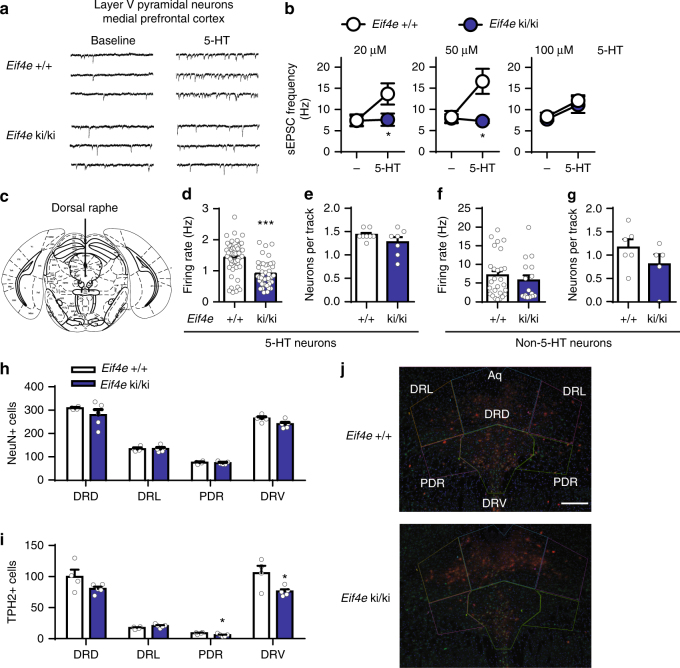
Serotonergic dysfunction in *Eif4e* ki/ki mice. **a** Representative whole cell recordings of spontaneous excitatory post-synaptic currents (sEPSCs) from layer V pyramidal neurons of the medial prefrontal cortex (mPFC) of wild-type and *Eif4e* ki/ki mice. Traces represent consecutive recordings of synaptic currents before (baseline) and after 5-HT (20 μM) treatment from single pyramidal cells in each group. **b** Frequency of sEPSCs from pyramidal neurons of wild-type and *Eif4e* ki/ki mice before (−) and after 5-HT at 20 μM (*n* = 7 cells from 5 *Eif4e*^+/+^; *n* = 8 cells from 7 *Eif4e* ki/ki), 50 μM (*n* = 4 cells from 3 *Eif4e*^+/+^; *n* = 5 cells from 3 *Eif4e* ki/ki) or 100 μM (*n* = 5 cells from 3 *Eif4e*^+/+^; *n* = 6 cells from 3 *Eif4e* ki/ki). **c** In vivo single-unit extracellular recordings of dorsal raphe neurons were performed in anesthetized *Eif4e*^+/+^and ki/ki mice. **d** Firing rate of dorsal raphe 5-HT neurons, measured in *Eif4e*^+/+^and ki/ki mice (*n* = 56 single unit recordings from 8 *Eif4e*^+/+^mice; *n* = 43 single unit recordings from 7 *Eif4e* ki/ki mice). **e** 5-HT neurons recorded per descent (track) in the dorsal raphe of wild-type and *Eif4e* ki/ki mice (*n* = 8 *Eif4e*^+/+^mice; *n* = 7 *Eif4e* ki/ki mice). **f** Firing rate of non-5-HT neurons in the DR (*n* = 27 single unit recordings from 6 *Eif4e*^+/+^mice; *n* = 16 single unit recordings from 5 *Eif4e* ki/ki mice). **g** Number of non-5-HT neurons per descent in the DR (*n* = 6 *Eif4e*^+/+^mice; *n* = 5 *Eif4e* ki/ki mice). **h** Number of NeuN + cells in the DR nuclei, which was subdivided into DR dorsal part (DRD), DR lateral part (DRL), posterodorsal raphe nucleus (PRN), and DR ventral part (DRV). Data are means of 4 slides per animal (*n* = 4 *Eif4e*^+/+^mice; *n* = 5 *Eif4e* ki/ki mice). **i** Number of tryptophan hydroxylase 2 (TPH2) positive cells in the DRN. **j** Representative images of the DR IHC; blue: nuclear DAPI staining; red: TPH2; green: NeuN. Scale bar is 100 μM. * *p* < 0.05, *** *p* < 0.001 vs. wild-type littermates (see Supplementary Table 2 for detailed results of statistical tests)

To understand the synaptic mechanisms underlying the depression-like state caused by inhibition of eIF4E phosphorylation, we first focused on serotonin (5-HT) neurotransmission in the medial prefrontal cortex (mPFC), a brain region central in depression pathophysiology^28–30^. Using whole-cell recording from layer V pyramidal neurons, we found no difference in the frequency of spontaneous excitatory postsynaptic current (sEPSCs) between wild-type and *Eif4e* ki/ki mice in baseline conditions (Fig. 2a,b). 5-HT stimulates sEPSCs in layer V pyramidal neurons^31^, mediated by excitatory 5-HT_2A_ postsynaptic receptors^32–34^. 5-HT (20 μM) augmented the frequency of sEPSCs in wild type by 84.55 ± 24.09 %, but not in *Eif4e* ki/ki mice (Fig. 2a, b). The NMDA-blocker and rapidly-acting antidepressant, ketamine, enhances 5-HT-induced sEPSCs^35^. We therefore tested whether the enhancement occurs in *Eif4e* ki/ki mice. After a single injection of ketamine (IP, 10 mg/kg) 24 h prior to recording, 5-HT increased the frequency of sEPSCs by 336.80 ± 64.88% over baseline in wild-type but not in *Eif4e* ki/ki mice (Supplementary Fig. 4a, b). In either condition, there were no changes in sEPSC rise and decay time (Supplementary Fig. 2b and 4d, e), and amplitude (Supplementary Fig. 2a and 4c), except for an increase in sEPSC decay time by 5-HT in saline-treated *Eif4e* wild-type and ki/ki mice (Supplementary Fig. 2c). To determine whether a greater dose of 5-HT would stimulate sEPSC in *Eif4e* wild type and ki/ki mice, we treated layer V mPFC slices with 50 and 100 μM of 5-HT. While both doses were excitatory in control mice, only the 100 μM dose induced an increase in sEPSC frequency as compared to that of control mice (Fig. 2b and Supplementary Fig. 2d, e). These results indicate that the depression-like phenotypes in *Eif4e* ki/ki mice are associated with decreased sensitivity to 5-HT excitatory neurotransmission in layer V mPFC.

Serotonergic neurons that innervate the mPFC reside in the dorsal raphe (DR) nuclei^36^. We thus determined whether the activity of DR neurons is affected in *Eif4e* ki/ki mice. To this end, we performed in vivo single-unit extracellular recordings of DR neurons^37^ (Fig. 2c). Male and female *Eif4e* ki/ki had a 36.19 ± 6.54 and 40.87 ± 16.27% decrease, respectively, in the firing rate of electrophysiologically defined 5-HT neurons compared to control littermates, respectively (Fig. 2d and Supplementary Fig. 3c), in the absence of changes in the number of spontaneously active neurons (neurons per descent; Fig. 2e and Supplementary Fig. 3d). Furthermore, the firing rate and number of non-5-HT neurons in DR was the same between genotypes (Fig. 2f, g). To determine whether the decreased firing rate of DR neurons was due to increased activity of the 5-HT_1A_ inhibitory autoreceptor^38^, DR neurons were recorded at baseline and following the administration of the 5-HT_1A_ antagonist, WAY-100635 (IP, 0.1-0.3 mg/kg). The latter induced a similar dose-dependent increase in DR neuron firing rate in wild-type and *Eif4e* ki/ki mice (Supplementary Fig. 3a, b), suggesting that the 5-HT_1A_ autoreceptors do not contribute to the diminished spontaneous activity of DR neurons in *Eif4e* ki/ki mice. These results indicate that reduction of eIF4E phosphorylation impaired serotonergic DR neuron firing and 5-HT post-synaptic excitation in the mPFC, in a manner similar to depression-inducing chronic stress^31,39^.

To determine whether the number of DR 5-HT neurons was altered in *Eif4e* ki/ki mice, we used tryptophan hydroxylase 2 (TPH2) as a histological marker. The number of TPH2 + cells was decreased in two subregions of the DR, the ventral part (DRV) and the posterodorsal raphe nucleus (PDR, Fig. 2h, j), while the total number of neurons (NeuN + cells) was the same between genotypes (Fig. 2i, j). When expressed as percent of the total neuronal population, the difference was only evident in the PDR (10.71 ± 1.039 vs 6.637 ± 1.179 % of NeuN + cells in PDR), suggesting a restricted reduction in 5-HT neurons in *Eif4e* ki/ki mice. These results are in agreement with the unchanged number of spontaneously active 5-HT neurons observed in in vivo electrophysiology (Fig. 2e and Supplementary Fig. 3d).

**Fig. 3:**
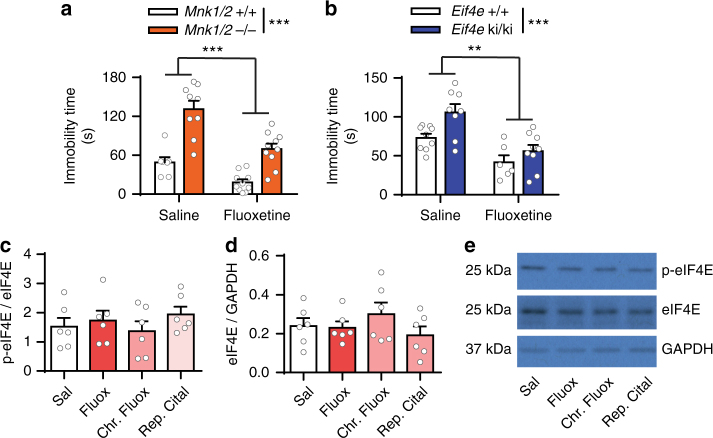
Normal response to Fluoxetine in *Eif4e* ki/ki mice. **a** Male wild-type and *Mnk1/2*^−/−^ mice were treated with saline or fluoxetine (IP, 3 mg kg^−1^) and tested in the FST after 30 min (*n* = 7 *Mnk1/2*^+/+^Saline, *n* = 9 *Mnk1/2*^−/−^ Saline; *n* = 10 *Mnk1/2*^+/+^Fluoxetine, *n* = 10 *Mnk1/2*^−/−^ Fluoxetine). **b** Male *Eif4e*^+/+^ and ki/ki were also treated with saline or fluoxetine (IP, 3 mg kg^−1^) and performed the FST after 30 min (*n* = 9 *Eif4e*^+/+^Saline, *n* = 8 *Eif4e* ki/ki Saline; *n* = 6 *Eif4e*^+/+^Fluoxetine, *n* = 9 *Eif4e* ki/ki Fluoxetine). **c** eIF4E phosphorylation in the mPFC/HPC of male wild-type mice treated with saline, 1 dose of fluoxetine (0.5 h, IP, 3 mg kg^−1^), chronic fluoxetine (14 × , IP, 10 mg kg^−1^) or repeated citalopram (3 × , IP, 10 mg kg^−1^) (*n* = 6/group). **d** Total eIF4E levels in the same samples as in (**d**). **e** Representative western blots for mice treated with vehicle or SSRIs. ** *p* < 0.01, *** *p* < 0.001 vs wild-type (see Supplementary Table 2 for detailed results of statistical tests)

The finding of impaired serotonergic neurotransmission prompted us to investigate the behavioral response of *Mnk1*/2^−/−^ and *Eif4e* ki/ki mice to the SSRI, fluoxetine. Acute treatment with fluoxetine (IP, 3 mg kg^−1^, 30 min) induced a significant decrease in immobility in the FST in both *Mnk1/2*^−/−^ and their wild-type littermates by 46.88 ± 9.52 % and 62.83 ± 27.23 %, respectively (Fig. 3a). Fluoxetine also reduced immobility in *Eif4e*^+/+^and ki/ki mice by 43.00 ± 16.85 % and 47.03 ± 10.75 %, respectively (Fig. 3b). Importantly, neither acute (IP, 3 mg kg^−1^, 0.5 h) nor chronic (IP, 14 × 10 mg kg^−1^) fluoxetine, or repeated (IP, 3 × , 10 mg kg^−1^) citalopram treatment changed the phosphorylation of eIF4E in the brain of wild-type mice (Fig. 3c, e). Similarly, ketamine treatment (IP, 10 mg kg^−1^, 1 h) did not change eIF4E phosphorylation in wild-type mice (Supplementary Fig. 4g–i), although it reduced immobility in the FST in both *Eif4e* ki/ki and their wild-type littermates (Supplementary Fig. 4f). These results indicate that these treatments do not engage the MNK1/2-eIF4E signaling pathway to induce anti-depressant-like effects.

We also determined that adult hippocampal progenitor proliferation (Ki67 + cells, Supplementary Fig. 5a, b), survival (BrdU + cells, 28 d after treatment, Supplementary Fig. 5c, d) and differentiation (DCX + cells, Supplementary Fig. 5e, f) was not changed in male and female *Eif4e* ki/ki mice.

### Inhibition of MNK1/2 induces depression-like phenotypes

**Fig. 4:**
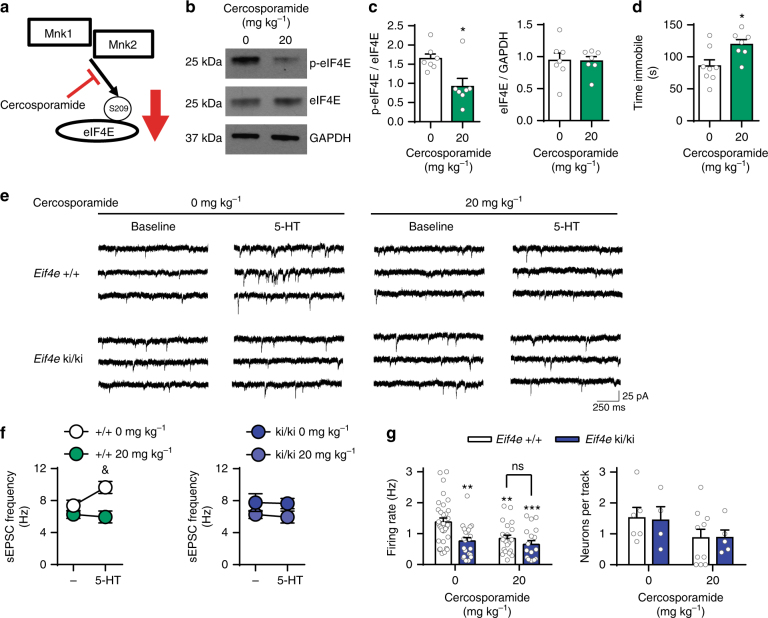
Pharmacological inhibition of MNK1/2 induces despair-like behavior and 5-HT synaptic impairments. **a** Cercosporamide, an MNK1 and MNK2 inhibitor, was administered for 5 consecutive days to wild-type mice (IP, 20 mg kg^−1^). **b** Representative western blot images for phosphorylated eIF4E (p-eIF4E), total eIF4E and GAPDH in the cortex-hippocampus of mice treated with vehicle or cercosporamide. **c** Average levels of p-eIF4E in vehicle and cercosporamide-treated wild-type mice (vehicle (0 mg kg^−1^), *n* = 7; cercosporamide, *n* = 7). **d** Cercosporamide administration induced increased immobility in the Forced Swim Test (FST) in wild-type mice (vehicle (0 mg kg^−1^), *n* = 8; cercosporamide, *n* = 7). **e** Representative sEPSC traces from recordings in layer V mPFC from wild-type or *Eif4e* ki/ki mice treated with vehicle or cercosporamide. **f** Layer V mPFC sEPSC frequency of wild-type (left panel, vehicle *n* = 5 neurons from three mice, cercosporamide *n* = 6 neurons from two mice) or *Eif4e* ki/ki mice (right panel, vehicle *n* = 5 neurons from 2 mice, cercosporamide *n* = 7 neurons from 3 mice) treated with vehicle or cercosporamide. **g** Firing rate of 5-HT DR neurons in wild-type and *Eif4e* ki/ki mice treated with vehicle or cercosporamide (*Eif4e*^+/+^: Vehicle 33 neurons from 6 mice, cercosporamide 22 neurons from 10 mice; *Eif4e* ki/ki: vehicle 22 neurons from 4 mice, cercosporamide 18 neurons from 4 mice). * *p* < 0.05, ** *p* < 0.01, ***p < 0.001 vs vehicle-treated wild-type mice; & *p* < 0.05 vs. saline-treated *Eif4e*^+/+^ (see Supplementary Table 2 for detailed results of statistical tests)

To rule out developmental/compensatory mechanisms in the depression- and anxiety-like behaviors in *Mnk1/2*^−/−^ and *Eif4e* ki/ki mice, we examined whether the depression-like behavior could be induced by pharmacological inhibition of MNK1 and 2 in adult wild-type mice using cercosporamide, an inhibitor of MNK1 and MNK2^40^ (Fig. 4a). Systemic administration of cercosporamide (IP, 5 × 20 mg kg^−1^^41^) decreased eIF4E phosphorylation in the brain by 44.18 ± 14.76 % (Fig. 4b, c), and enhanced immobility in the FST by 33.49 ± 12.66 s (Fig. 4d).

Furthermore, in wild-type mice cercosporamide also inhibited the excitatory response of 5-HT in layer V mPFC pyramidal neurons (Fig. 4e, f) and decreased the firing rate of 5-HT DR neurons (Fig. 4g). Importantly, cercosporamide failed to change any of these parameters in *Eif4e* ki/ki mice (Fig. 4e, g), indicating an effect through eIF4E phosphorylation and not via unknown MNK1/2 targets. These results strongly support a direct role of eIF4E phosphorylation in the pathophysiology of depression.

### Dysregulated TNFα synthesis in the brain of Eif4e ki/ki mice

**Fig. 5:**
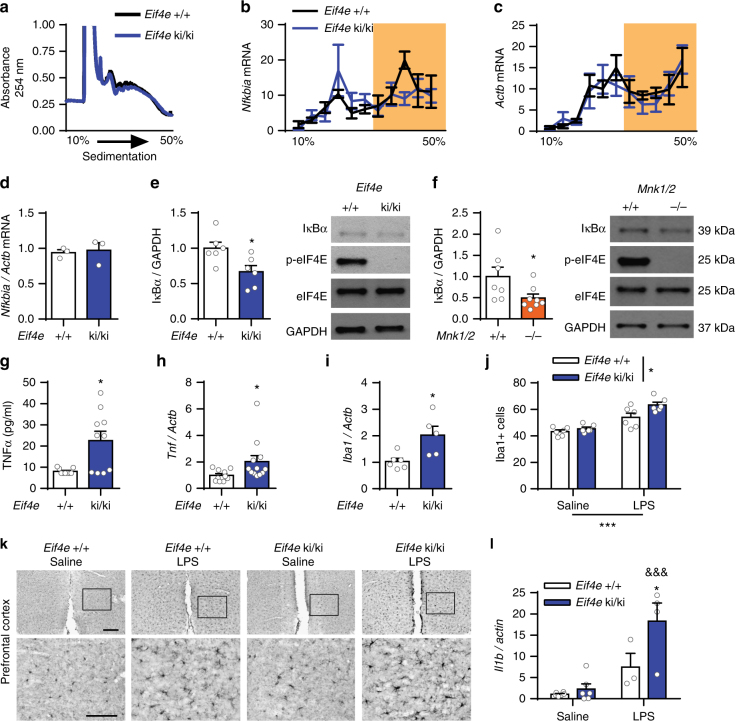
Translation of *Nfkbia* mRNA is reduced in *Eif4e* ki/ki mice. **a** Representative polysome profile of cortex-hippocampus from *Eif4e*^+/+^and ki/ki mice (1 mouse per genotype). **b** Distribution of the *Nfkbia* mRNA in the polysome fractions of wild-type and *Eif4e* ki/ki mice (*n* = 4 *Eif4e*^+/+^; *n* = 5 *Eif4e* ki/ki). **c**, *Actb* mRNA was measured in both mouse strains as a control. **d** Total mRNA levels of *Nfkbia* in the cortex-hippocampus of *Eif4e*^+/+^and ki/ki mice. **e** Average protein levels and representative western blot images of the *Nfkbia* mRNA product, IκBα, in the mPFC of *Eif4e* wild-type and ki/ki mice (*n* = 6/group). **f** Average protein levels and representative western blot images IκBα, in the mPFC of *Mnk1/2*^+/+^ and ^−/−^ mice (*n* = 7 *Mnk1/2*^+/+^; *n* = 8 *Mnk1/2*^−/−^). **g** Levels of the cytokine tumor necrosis factor α (TNFα) in the mPFC in wild-type and *Eif4e* ki/ki mice (*n* = 9 *Eif4e*^+/+^; *n* = 10 *Eif4e* ki/ki). **h** Total levels of *Tnf* mRNA in the mPFC in wild-type and *Eif4e* mutant mice (*n* = 10 *Eif4e*^+/+^; *n* = 12 *Eif4e* ki/ki). **i** mRNA levels of the microglial marker Iba1 (*Aif1*) in the mPFC in wild-type and *Eif4e* mutant mice (*n* = 6 *Eif4e*^+/+^; *n* = 5 *Eif4e* ki/ki). **j** Number of Iba1 positive cells (Iba1+) per 0.01 mm^2^ of mPFC from wild-type and *Eif4e* ki/ki mice 48 h after saline or LPS (IP, 2.5 mg kg^−1^) treatment (*n* = 3/group). **k** Representative images of the mPFC of wild-type or *Eif4e* ki/ki mice 48 h after saline or LPS treatment. **l** mRNA levels of *Il1b* in the mPFC of wild-type or *Eif4e* ki/ki mice 48 h after saline or LPS treatment (*n* = 6 *Eif4e*^+/+^Saline, n = 6 *Eif4e* ki/ki Saline, *n* = 3 *Eif4e*^+/+^LPS; *n* = 4 *Eif4e* ki/ki LPS). * *p* < 0.05, *** *p* < 0.001 vs. wild-type; &&& *p* < 0.001 vs. saline-treated *Eif4e* ki/ki (see Supplementary Table 2 for detailed results of statistical tests)

To gain insight into the molecular mechanism by which the lack of eIF4E phosphorylation results in depression-like behavior and altered serotonergic neurotransmission, we inspected the list of mRNAs whose translation is significantly reduced in the absence of eIF4E phosphorylation^12^. Among these, the *Nfkbia* mRNA, encoding for the negative regulator of NF-κB, IκBα, is of particular interest^12,42^ because of the prominent role of inflammatory mechanisms in the pathophysiology of MDD and impaired serotonergic neurotransmission^19,21^. Translation initiation of the *Nfkbia* mRNA was determined based on its association with polysomes using sucrose density gradients (Fig. 5a). In *Eif4e* ki/ki mice, the association of *Nfkbia* mRNA with the heavy polysome fractions is decreased in cortico-hippocampal tissue (including mPFC), consistent with a reduction in the translation of this mRNA in the brain (Fig. 5b). As a control, the distribution of the *Actb* mRNA was not changed (Fig. 5c), and total levels of *Nfkbia* mRNA were the same in control versus *Eif4e* ki/ki mice (Fig. 5d). To demonstrate that the altered polysome profile of the *Nfkbia* mRNA resulted in reduced amounts of IκBα protein, we used Western blotting to measure its levels in the mPFC. In *Eif4e* ki/ki and *Mnk1/2*^−/−^ mice, IκBα levels were decreased (by 50.88 ± 23.11 % and 33.33 ± 12.44 %, respectively) as compared to their corresponding littermate controls (Fig. 5e, f). Taken together, these results demonstrate dysregulated mRNA translation of IκBα in the mPFC upon inhibition of eIF4E phosphorylation.

To investigate the pathophysiological consequence of the decrease in IκBα, we used a multiplex immunoassay to determine the baseline levels of 23 cytokines, which are known to be regulated by IκBα/NF-κB, from the PFC of *Mnk1/2*^−/−^ and *Eif4e* ki/ki mice (Supplementary Table 1). Only eotaxin and tumor necrosis factor α (TNFα) appeared increased in the PFC of both, *Mnk1/2*^−/−^ and *Eif4e* ki/ki mice. We could not detect a difference in eotaxin levels from independent PFC samples using ELISA (Supplementary Fig. 6a), although it was reduced in plasma samples of *Mnk1/2*^−/−^ and *Eif4e* ki/ki mice (Supplementary Fig. 6b). In contrast, TNFα levels were increased in the PFC of *Eif4e* ki/ki and *Mnk1/2*^−/−^ mice by 101.9 ± 42.75% and 178.34 ± 55.33 % over controls, respectively (Fig. 5g and Supplementary Fig. 6c). Consistent with transcriptional regulation of *Tnf*, its mRNA levels were increased in the PFC of *Eif4e* ki/ki by 104.3 ± 46.83 % over control levels (Fig. 5h). In the circulation, TNFα levels were elevated only in *Mnk1/2*^−/−^ (by 203.8 ± 91.62 %), but not in *Eif4e* ki/ki mice (Supplementary Fig. 6d, e). Furthermore, levels of the soluble TNF receptor 1 (sTNFR1) were induced in the serum of *Eif4e* ki/ki mice (Supplementary Fig. 6f). sTNFR1 stabilizes TNFα and its biological activity, thus promoting inflammation^43^.

Next, we investigated whether increased TNFα led to neuroinflammation. To this end, we measured the levels of *Iba1* (*Aif1*) mRNA, a marker of microglial activation^44^. We observed increased levels of * Iba1* in the PFC (99.02 ± 33.31 % over control) (Fig. 5i), and in the hippocampus (100.28 ± 34.73 % over control) of *Eif4e* ki/ki mice (Supplementary Fig. 6g). Next, we challenged *Eif4e*^+/+^and ki/ki mice with the immunogen, lipopolysaccharide (LPS, IP, 2.5 mg kg^−1^,), and analyzed the brains of the mice 48 h later; a timeline whereby acute inflammatory responses have receded, but depression-like behaviors persist^45^. *Eif4e* ki/ki mice had more Iba1 + cells in the prefrontal cortex (Fig. 5j, k), and the hippocampus (Supplementary Fig. 6h, i) following both saline and LPS treatments. Furthermore, *Il1b* mRNA was induced to greater levels in the *Eif4e* ki/ki mice compared to wild-type counterparts 48 h after LPS stimulation (145.42 ± 47.84% over *Eif4e*^+/+^treated with LPS) (Fig. 5l). Taken together, these results demonstrate that inhibition of eIF4E phosphorylation causes reduced translation of the *Nfkbia* mRNA, leading to increased levels of TNFα expression and an inflammatory state in the brain.

### TNFα mediates depression-like phenotypes in Eif4e ki/ki mice

**Fig. 6:**
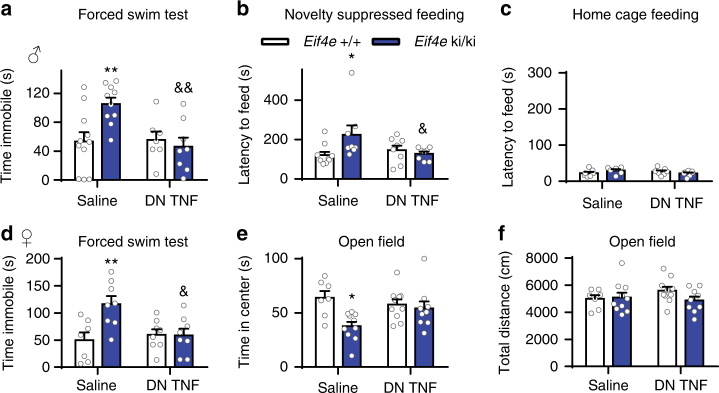
TNFα causes behavioral impairments in *Eif4e* ki/ki mice. **a** wild-type and *Eif4e* ki/ki mice were administered (ICV) a dominant negative mutant version of TNFα (DN TNF) or vehicle for 12 days (via an osmotic pump connected to a guide cannula). Male mice were then evaluated in the FST (*n* = 11 *Eif4e*^+/+ ^Saline, *n* = 7 *Eif4e*^+/+ ^DN TNF, *n* = 10 *Eif4e* ki/ki Saline, *n* = 8 *Eif4e* ki/ki DN TNF). **b** Mice were also tested in the novelty suppressed feeding task (*n* = 10 *Eif4e*^+/+ ^Saline, *n* = 8 *Eif4e*^+/+ ^DN TNF, *n* = 8 *Eif4e* ki/ki Saline, *n* = 8 *Eif4e* ki/ki DN TNF). **c** Latency to feed in their home cage was also measured. **d** Female mice also administered with vehicle or DN TNF (ICV) were evaluated in the FST (*n* = 7 *Eif4e*^+/+ ^Saline, *n* = 8 *Eif4e*^+/+ ^DN TNF, *n* = 8 *Eif4e* ki/ki Saline, *n* = 8 *Eif4e* ki/ki DN TNF). **e** Female mice exploratory behavior was also measured in the center of an open field (OF) (*n* = 7 *Eif4e*^+/+ ^Saline, *n* = 10 *Eif4e*^+/+ ^DN TNF, *n* = 10 *Eif4e* ki/ki Saline, *n* = 9 *Eif4e* ki/ki DN TNF). **f** Their locomotion in the OF was also measured. * *p* < 0.05, ** *p* < 0.01, *** *p* < 0.001 vs. wild-type saline; &, *p* < 0.05, && *p* < 0.01, &&& *p* < 0.001 vs. saline-treated *Eif4e*^+/+^or ki/ki (see Supplementary Table 2 for detailed results of statistical tests)

To investigate the contribution of the elevated brain levels of TNFα in *Eif4e* ki/ki and *Mnk1*/2^−/−^ mice towards the behavioral and serotonergic alterations, we treated mice with a dominant negative mutant of TNFα (DN TNF, XPro1595^46^). Central administration of DN TNF (ICV, 12 days, 10 mg ml^−1^ in 100 μl, 0.25 μl h^−1^) effectively reduced the increased immobility in the FST for the *Mnk1/2*^−/−^ (Supplementary Fig. 7a) and *Eif4e* ki/ki mice (Fig. 6a) by 48.78 ± 13.59 and 59.20 ± 16.69 s, respectively, to levels indistinguishable from saline-treated control mice. DN TNF also reduced immobility in female *Eif4e* ki/ki by 57.59 ± 17.98 s (Fig. 6d). Importantly, the treatment did not affect FST immobility in wild-type littermates (Supplementary Fig. 7a and Fig. 6a, d). Furthermore, centrally administered DN TNF decreased the incremented latency to feed in the NSF test in *Mnk1/2*^−/−^ (Supplementary Fig. 7b) and *Eif4e* ki/ki mice (Fig. 6b), by 268.0 ± 75.31 and 95.50 ± 39.72 s, respectively, to levels similar to wild-type controls. The latency to feed in the home cage was not affected by the DN TNF treatment in neither wild-type nor mutant mice (Supplementary Fig. 7c and Fig. 6c). Decreased exploration of the center of the open field was increased in female *Eif4e* ki/ki mice (Fig. 6e), but not general locomotion (Fig. 6f).

**Fig. 7:**
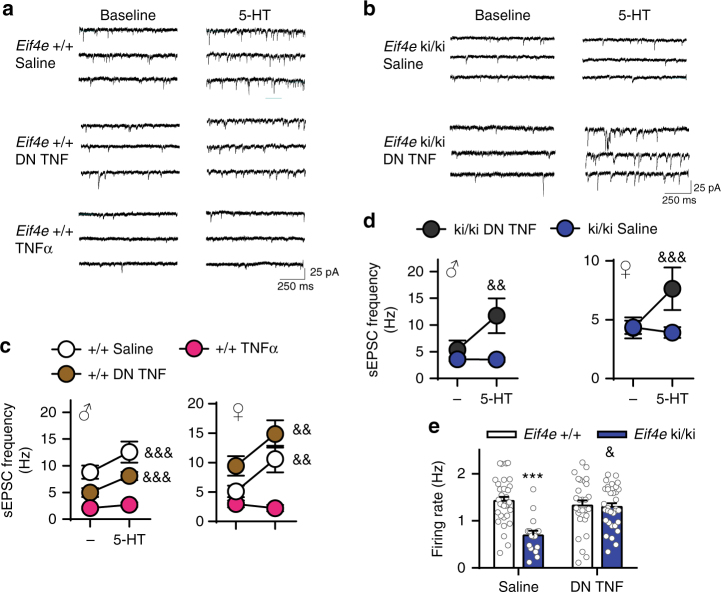
TNFα causes serotonergic impairments in *Eif4e* ki/ki mice. **a** Representative sEPSC traces from mPFC pyramidal neurons in brain slices of wild-type mice treated with vehicle (saline), DN TNF (200 ng ml^−1^) or mouse recombinant TNFα (10 ng ml^−^^1^). Synaptic currents were recorded before (baseline) and after 5-HT (20 μM) administration. **b** Representative sEPSC traces from *Eif4e* ki/ki mice treated with either vehicle or DN TNF (200 ng ml^−1^), measured before and after 5-HT (20 μM) administration. **c** Frequency of sEPSCs in mPFC slices of male and female wild-type mice. Slices were directly treated with saline (M: *n* = 10 cells from 6 mice; F: *n* = 9 cells from 6 mice), DN TNF (200 ng ml^−1^; M: *n* = 8 cells from 5 mice; F: *n* = 7 cells from 4 mice) or mouse recombinant TNFα (10 ng ml^−1^; M: *n* = 9 cells from 6 mice; F: *n* = 6 cells from 5 mice), pre- and post-5-HT (20 μM) application. **d** Average sEPSC frequency in *Eif4e* ki/ki mice before and after 5-HT (20 μM) application; slices were treated with either saline (M: *n* = 11 cells from 6 mice; F: *n* = 9 cells from 7 mice) or DN TNF (200 ng ml^−1^; M: *n* = 8 cells from 6 mice; F: *n* = 9 cells from 7 mice). **e**, Firing rate of DR neurons in wild-type and *Eif4e* ki/ki mice chronically treated with DN TNF or saline (ICV, 12 days; *n* = 33 recordings from 6 *Eif4e*^+/+ ^Saline mice; *n* = 31 recordings from 5 *Eif4e*^+/+ ^DN TNF mice; *n* = 16 recordings from *Eif4e* ki/ki Saline mice; *n* = 33 recordings from 6 *Eif4e* ki/ki DN TNF mice). * *p* < 0.05, ** *p* < 0.01, *** *p* < 0.001 vs. wild-type saline; & *p* < 0.05, && *p* < 0.01, &&& *p* < 0.001 vs. saline-treated *Eif4e*^+/+^or ki/ki (see Supplementary Table 2 for detailed results of statistical tests)

Moreover, 5-HT-induced excitatory synaptic activity in layer V pyramidal neurons was restored in male and female *Eif4e* ki/ki mice following direct treatment with DN TNF (200 ng ml^−1^, 1 h incubation) of the mPFC slices (Fig. 7a–c). The same treatment did not change the 5-HT-induced increase in sEPSC frequency (Fig. 7a, c) or amplitude (Supplementary Fig. 2g) in wild-type mice. Finally, in vivo treatment with DN TNF restored the firing rate of the DR neurons in *Eif4e* ki/ki mice to control levels in vivo (Fig. 7e).

**Fig. 8:**
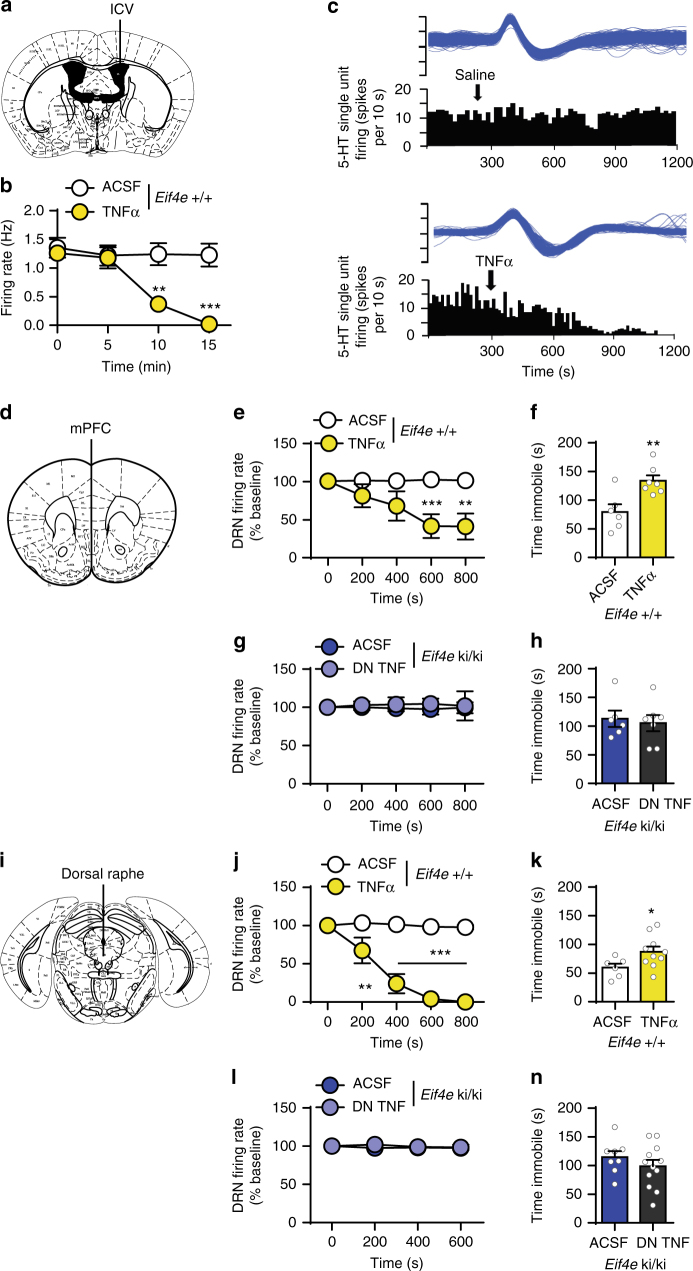
Simultaneous inhibition of mPFC and DR by TNFα underlies despair-like behavior. **a** Acute recombinant mouse TNFα (ICV, 0.02 pg ml^−1^) into wild-type mice. **b** Firing rate of DR neurons in wild-type mice treated with either artificial cerebrospinal fluid (ACSF) (*n* = 4) or mouse recombinant TNFα (*n* = 6) averaged in 5 minute bins. **c** Representative integrated firing rate histograms (spikes per 10 s) showing traces of DR neurons in wild-type mice treated with either saline or mouse recombinant TNFα. **d** Acute treatment of TNFα (0.1 fg in 0.5 µl ACSF) into the mPFC of wild-type mice or DN TNF (100 pg in 0.5 μl ACSF) to *Eif4e* ki/ki mice. **d** Firing rate of DRN 5-HT neurons of wild-type mice treated with ACSF (*n* = 6) or TNFα (*n* = 6) into the mPFC. **e** Immobility time in the FST, 20 min after ACSF (*n* = 6) or TNFα (*n* = 7) into the mPFC of wild-type mice. **f**, Firing rate of DRN 5-HT neurons of *Eif4e* ki/ki mice treated with ACSF (*n* = 5) or DN TNF (*n* = 5) into the mPFC. **g** FST immobility time 20 min after ACSF (*n* = 6) or DN TNF (*n* = 7) into the mPFC of *Eif4e* ki/ki mice. **h** Similar treatments were performed into the DRN. **i** 5-HT DR neuron firing rate after ACSF (*n* = 7) or TNFα (*n* = 6) in wild-type mice. **j** Despair-like behavior in wild-type mice treated with ACSF (*n* = 6) or TNFα (*n* = 10). **k** DRN 5-HT neurons in *Eif4e* ki/ki treated with ACSF (*n* = 5) or DN TNF (*n* = 4). **l** FST immobility in *Eif4e* ki/ki treated with ACSF (*n* = 8) or DN TNF (*n* = 12). * *p* < 0.05, ** *p* < 0.01, *** *p* < 0.001 vs. wild-type ACSF (see Supplementary Table 2 for detailed results of statistical tests)

To demonstrate that TNFα causes deficits in 5-HT neurotransmission, as it does for behavior^47^, we determined the 5-HT response of layer V PFC neurons in wild-type mice following direct TNFα treatment to the slices (10 ng ml^−1^, 1 h). TNFα treatment ameliorated the 5-HT-induced increase in sEPSC frequency in male and female mice (Fig. 7a, c) without affecting their amplitude (Supplementary Fig. 2g). In vivo, an acute treatment with TNFα (ICV, 0.02 fg µl^−1^) also elicited a rapid decrease in the frequency of spontaneous firing rate of DR neurons in wild-type mice (Fig. 8a–c). These results support the conclusion that over-expression of TNFα is necessary and sufficient to induce depression-like behavior and impaired serotonergic neurotransmission as a consequence of decreased eIF4E phosphorylation.

To better understand the link between TNFα-induced deficits in 5-HT neurotransmission in DR and mPFC, we determined the effects of acute TNFα or DN TNF treatments in the mPFC (Fig. 8d) on despair-like behavior and DR firing. Acute infusion of TNFα in the mPFC of wild-type mice (0.1 fg in 0.5 µl of ACSF) rapidly induced immobility in the FST (Fig. 8f) and a decreased firing of DR 5-HT neurons (Fig. 8e). However, acute DN TNF administration to the mPFC of *Eif4e* ki/ki mice (100 pg in 0.5 μl of ACSF), which restores the 5-HT-induced sEPSC excitation in the mPFC (Fig. 7b, d), failed to rescue the immobility in the FST (Fig. 8h) and did not influence the DR 5-HT firing (Fig. 8g). Finally, acute TNFα infusion in the DR (Fig. 8i) of wild-type mice, also induced immobility in the FST (Fig. 8k) and decreased DR 5-HT neuronal firing (Fig. 8j). Like treatment to the mPFC, acute DN TNF microinjection to the DR did not rescue the increased FST (Fig. 8n) nor did it diminish the firing rate of DR 5-HT neurons in *Eif4e* ki/ki mice (Fig. 8l). These results show that in the *Eif4e* ki/ki mice, TNFα simultaneously targets the mPFC and DR to engender the decrease in function of the bidirectional mPFC-DR circuit^48^, leading to despair-like behavior.

## Discussion

We demonstrated that in mice, a deficit in eIF4E phosphorylation triggers depression and anxiety phenotypes (Figs. 1, 4 and Supplementary Fig. 1), as well as reduced activity of DR neurons and impaired serotonin neurotransmission in the PFC (Figs. 2, 4 and Supplementary Fig. 3 and 4). These effects coincided with a reduction in brain levels of the NF-κB inhibitor, IκBα, leading to over production of TNFα, and hypersensitivity to the sequelae of inflammatory stimuli (Fig. 5 and Supplementary Fig. 6). Brain-wide blocking of TNFα diminished depressive behaviors and rescued the impairment in serotonergic neurotransmission (Figs. 6, 7 and Supplementary Fig. 7). Based on these findings, we offer a model (Supplementary Fig. 8) whereby reduction of eIF4E phosphorylation contributes to the development of depressive symptoms and 5-HT dysfunction by modulating the inflammatory response through the translational control of IκBα expression.

Clinical and preclinical data support a strong link between inflammation and mood disorders. First, the levels of cytokines, including TNFα and IL-6, are increased in MDD patients^22,23^, as well as in individuals that have suffered childhood trauma and abuse^49^, a well-documented risk factor for MDD and other mental disorders^50^. Second, these observations have been replicated in rodent models of depression induced by chronic stress^51,52^. Third, infection-triggered sickness behaviors, which are similar to symptoms of depression, can be treated with classical antidepressants^45,53^. Fourth, clinical trials suggest that non-steroidal anti-inflammatory drugs (NSAIDs) are beneficial for depression^54^, as well as anti-cytokine therapies, including those using blocking antibodies to target TNFα^55^.

Our findings support the important role that the NF-κB pathway plays in the pathophysiology of depressive phenotypes through the control of serotonergic neurotransmission^19^. In this regard, chronic stress can also increase the sensitivity of NF-κB activation to inflammatory stimuli in the PFC and hippocampus. This occurs via the glucocorticoid receptor, and can be reversed by MK-801^56^, an NMDA receptor antagonist with anti-depressant activity like ketamine^57^. Furthermore, the IκB Kinase (IKK) in the nucleus accumbens (NAc) plays an important role in controlling mood^58^ and mediating the effects of chronic social stress in synaptic morphology and in the behavioral expression of social defeat^59^. IKKs lie directly upstream of IκBα and are responsible for its phosphorylation and degradation, leading to NF-κB activation and nuclear translocation^60^. Activation of NF-κB by chronic stress is also responsible for the stress-induced decrease in neurogenesis in the hippocampus and anhedonia^61^. Importantly, in humans, it was reported that social stress increases NF-κB signaling in peripheral blood mononuclear cells of healthy subjects^62^ and this response is exaggerated in depressed patients^63^.

Targeting the NF-κB signaling pathway for the treatment of depression has the potential drawback of affecting a myriad of immune functions^60^, as well as impairing normal neuronal physiology and cognition^64,65^. In contrast, targeting TNFα would be more appealing in light of its role in the pathophysiology of depression, particularly deficits in serotonin neurotransmission^21^. A meta-analysis of clinical data suggests that blocking antibodies against TNFα are consistently effective in improving mood in patients with chronic inflammatory diseases independently from the outcome of their accompanying physical illness^55^.

While the current study was being considered for publication, a different research group reported depression-like behaviors and increased inflammatory responses in the *Eif4e* ki/ki mice^66^. Behavioral phenotypes in our *Eif4e* ki/ki mouse testing are consistent with those reported by reference^66^. In addition, our study demonstrated the involvement of MNK1 and MNK2 as the upstream kinases of eIF4E, and demonstrate the involvement of TNFα as a mediator of serotonergic neurotransmission impairment in the PFC and the DR, as a consequence of decreased eIF4E phosphorylation and attenuated translation of the mRNA encoding IκBα.

In summary, we demonstrated that decreased mRNA translation via inhibition of eIF4E phosphorylation regulates inflammatory responses via IκBα translation and induction of TNFα in mice, eliciting an inflammatory state, deficits in 5-HT neurotransmission and a depressive state that is clinically relevant to MDD.

## Methods

### Mice

Adult male and female mice (P60-180) were used in these studies. *Mnk1/2* double knockout and *Eif4e* ki/ki mice have been reported^11,12^. Mice were housed in groups of 2–5 per cage with ad libitum access to food and water (except in experiments involving food deprivation), in temperature (21 °C) and humidity (~55%) controlled rooms, and a 12, h light/dark cycle. All procedures followed the Canadian Council on Animal Care guidelines and were approved by McGill University and Université de Montréal Animal Care Committees.

### Behavioral procedures

Mice were handled once 24 h before each experiment. All behavioral experiments were performed between 10:00 am and 3:00 pm. Behaviors (except for the open field) were recorded, stored, and analyzed as MPEG files using an automated behavioral tracking system (Videotrack, View Point Life Science, Montreal, Canada) equipped with infrared lighting-sensitive CCD cameras. The analog signals supplied by the camera were measurements of the luminosity of each point of the image scanned point-by-point and line-by-line at a rate of 25 images per second. The signals were transmitted to the Videotrack system and digitized on 8 bits by digital analog conversion. Before the experiments, animal/image background contrast detection thresholds were calibrated by visual inspection to distinguish different behavioral patterns.

For the forced swim test, mice were placed in a glass beaker (24 cm tall, 14 cm diameter) containing 3 L of water at 24 ± 2°C. Mice were allowed to swim for 6 minutes. Immobility was quantified in the last 4 minutes of the test using the Videotrack system.

For the tail suspension test, mice were suspended by their tail from a lever in a 30 × 30 × 30 cm white-painted enclosure. Movements were recorded and quantified for 5 min in a dimly lit environment.

For the novelty suppressed feeding, mice were food-deprived for 48 h, then each mouse was placed in a brightly illuminated (100 W, 350 lx) open arena (40 × 40 white-painted floor with white walls 30 cm in height) containing lab chow (3 pellets) placed at the center of the arena. The latency to initiate feeding (in seconds) was noted and used as an index of anxiety/depression-like behavior. The cut-off time was 600 s. Feeding latency was also observed in the home cage containing 3 pellets spread on the floor. The session was terminated immediately after mice had initiated feeding.

Locomotor activity and time spent in the center of an open field were quantified for 10 min with an infrared activity-monitoring acrylic apparatus with activity sensors located on the sides, front, and back of the boxes (VersaMax system, AccuScan Instruments, Columbus, OH, U.S.A.). *Mnk1/2*^−/−^ and wild-type littermates were tested twice in the apparatus, with the second test occurring 1 week after the initial test.

### Anti-depressant treatments

Fluoxetine hydrochloride (Tocris Bioscience, Oakville ON, Canada), escitalopram oxalate (Tocris Biosceicne) and ketamine (Narketan 10, Vetoquinol, Lavaltrie, QC, Canada) were diluted in 0.9% NaCl solution and administered IP as follows: acute fluoxetine, 1 dose of 3 mg kg^‒1^ (behavioral testing/tissue collection occurred 0.5 h after injection); chronic fluoxetine, 14 doses (daily) of 10 mg kg^‒1^ (tissue collection occurred 0.5 h after last injection); repeated citalopram 3 doses of 10 mg kg^‒1^ (24, 5 and 0.5 h before tissue collection); ketamine (10 mg kg^‒1^) 1 or 24 h before behavioral testing/tissue collection or mPFC whole-cell recordings.

### Cercosporamide treatment

Intraperitoneal (i.p.) injections were carried out using an aqueous solution of 4% Tween 80 (Sigma-Aldrich) and 4% PEG-400 (Sigma-Aldrich). Cercosporamide was administered daily by i.p. injections for five consecutive days at 20 mg kg^‒1^ to 8 week-old C57BL/6 J male mice (Jackson Laboratory). Mice were tested in the FST 1 h after the last injection and immediately sacrificed for brain collection. Other cohorts of wild-type and *Eif4e* ki/ki mice were treated likewise and used for layer V mPFC whole-cell recordings or in vivo DR recordings, 1 h after the last injection.

### Mouse serum and brain collection

Naive mice (*Mnk1/2*^−/−^, *Eif4e* ki/ki and their respective wild-type littermates) were briefly anesthetized using 5% isofluorane. Blood was collected by heart puncture and allowed to clot for 10 min at 4 °C. Serum was separated by centrifugation (10 min, 4 °C, 5000 × *g*) and stored at −80 °C. Brains were harvested immediately after heart puncture, prefrontal cortex and hippocampus dissected, flash frozen and kept at −80 °C until used.

In a separate experiment, *Eif4e* ki/ki and wild-type littermates were treated with saline (IP, 10 ml kg^−1^) or LPS (E. Coli 0111:B4, 2.5 mg kg^−1^ ^45^, Sigma-Aldrich, St Louis MO, U.S.A.) and sacrificed 48 h later^45^. At that point, brains were harvested immediately, prefrontal cortex and hippocampus dissected, flash frozen and kept at −80 °C until used or processed for immunohistochemical analyses.

### Polysome profiling

Polysome profiling was performed as previously reported^67^. Briefly, hippocampi and prefrontal cortex were dissected and snap frozen, then homogenized in 425 μl of hypotonic buffer [(5 mM Tris-HCl (pH 7.5), 2.5 mM MgCl_2_, 1.5 mM KCl and 1 × protease inhibitor cocktail (EDTA-free)], with 5 μl of 10 mg/ml cycloheximide, 1 μl of 1 M DTT, and 100 units of RNAse inhibitor. After homogenization, 25 μl of 10 % Triton X-100 (final concentration 0.5 %) and 25 μl of 10 % Sodium Deoxycholate (final concentration 0.5 %) were added. Following a 10 min incubation at 4 °C, samples were centrifuged (16,000 × g for 7 min at 4 °C). After adjusting for RNA concentration, the supernatant was applied to a 5–50% sucrose gradient and centrifuged at 222,228 × g (36,000 rpm), for 2 hr at 4 °C. Gradients were fractionated using an automated collector (Teledyne ISCO Foxy R1 Fraction Collector, Cole Palmer) connected to a syringe pump (Brandel) and a UV detector (Teledyne ISCO UA, 6 UV/VIS Detector). RNA was extracted from the collected fractions, along with an input sample, using TRIzol reagent (Ambion by Thermo Fisher Scientific). cDNA was then synthesized using Super Script III reverse transcriptase (Invitrogen by Thermo Scientific) using 250 ng of RNA as template and then *Nfkiba* and *Actb* mouse specific TaqMan assays (Applied Biosystems by Thermo Fisher Scientific). mRNA levels were estimated using the ΔΔCt method.

### Bio-Plex Immunoassay

Flash-frozen prefrontal cortex from *Eif4e* ki/ki, *Mnk1/2*^−/−^ and their respective wild-type littermate control mice were lysed using the Bio-Plex Cell Lysis Kit (Bio-Rad, Mississauga, ON, Canada) following the manufacturer’s instructions for tissue samples. Samples were assayed in the Bio-Plex Pro mouse cytokine 23-plex assay following the manufacturer’s instructions. The plate was read in the Bio-Plex 200 plate reader.

### ELISA

Serum or prefrontal cortex lysate (using the Bio-Plex Cell Lysis Kit) were assayed for eotaxin or TNFα using Quantikine ELISAs for mouse eotaxine or TNFα (R&D Systems, Minneapolis, MN, U.S.A.) following the manufacturer’s instructions. Soluble TNF receptor 1 (sTNFRI) was also measured in mouse serum (Quantikine ELISA, R&D Systems, Minneapolis, MN, U.S.A.)

### Real-time qPCR

Flash-frozen prefrontal cortex and hippocampus were processed for RNA extraction using TRIzol reagent (Ambion by Thermo Fisher Scientific). cDNA was then synthesized using Super Script III reverse transcriptase (Invitrogen by Thermo Scientific) using 500 ng of total RNA as template and then *Tnf*, *Il1b*, *Iba1* (*Aif1*) and *Actb* mouse specific TaqMan assays (Applied Biosystems by Thermo Fisher Scientific). mRNA levels were estimated using the ΔΔCt method.

### Western Blotting

Mouse tissues were dissociated using Bio-Plex Cell Lysis Kit (Bio-Rad, Mississauga, ON, Canada). Western blotting was performed as previously described^68^. Antibodies against indicated proteins were: phospho-eIF4E (Ser209, Abcam, 1:1000), eIF4E (BD Biosciences, 1:1000), IκBα (Cell Signaling Technology, 1:1000), and glyceraldehyde 3-phosphate dehydrogenase (GAPDH, coupled to Horseradish Peroxidase, Abcam, 1:5000) or β-actin (Sigma, 1:5000); secondary antibodies were anti-mouse and anti-rabbit (GE Healthcare). Quantification of immunoblots was performed using ImageJ (NIH), and expressed as a ratio (either p-eIF4E/eIF4E or eIF4E/GAPDH or eIF4E/β-actin, IκBα/GAPDH). Western blots experiments were replicated at least two times. Uncropped western blots are provided in Supplementary Figure 9.

### Immunohistochemistry

For the analysis of neuroinflammation markers, coronal brain sections were thin cut (50 μm) using a Cryostat and placed in PBS containing 2 mM sodium azide and 3 mM NaF, pH 7.4. For the immunohistochemical staining, sections were first treated with 0.3% H_2_O_2_ and 20% methanol in PBS for 10 min to deactivate endogenous peroxidases and permeabilize the tissue, and then blocked for 1 h in 10% goat serum/PBS and incubated (overnight, 4 °C) in rabbit anti-IBA1 antibody (1:1000 final dilution). Next, tissue was incubated for 1.5 h at room temperature in biotinylated anti-rabbit IgG (1:200; Vector Laboratories, Burlingame, CA) and then placed in an avidin/biotin HRP complex for 1 h (prepared per instructions of the manufacturer; Vector Laboratories). Sections were washed in PBS (three times, 10 min/wash) between each labeling step. The signal was visualized using nickel-intensified DAB substrate (Vector Laboratories) and sections were mounted on gelatin-coated slides with Permount media (Fisher Scientific, Houston, TX).

Bright-field microscopy images were captured using a digital camera mounted on an inverted Zeiss microscope (Oberkochen, Germany). All data were quantified using Adobe Photoshop software (Adobe Systems Incorporated, San Jose, CA). For cell counting, an intensity threshold filter was initially applied to eliminate nonspecific background labeling, and then the number of detectable signals above the threshold (now defined as positive cells) were counted on the 100 × micrographs for the hippocampal CA1 area and the prefrontal cortex. A mean value for each animal was generated from 2 brain sections per animal; this value was then used to generate the group mean.

For the quantification of 5-HT and total number of DR neurons, coronal DR sections were fixed in 2% paraformaldehyde (10 min, RT) washed in PBS, and then incubated in 10% normal donkey serum (NDS) and 0.2% Triton X (1 h, RT). Then, primary antibodies were applied (in 10% NDS, 4 C for 48 h). Antibodies were: polyclonal rabbit anti-TPH2 (Novus Biologicals, 1:1000) and monoclonal mouse anti-NeuN (Millipore, 1:150). After washing, avidin/biotin blocking was performed (Avidin/Biotin blocking kit, Vector Laboratories), then slices were incubated with Cy3 donkey anti-rabbit (Linaris, 1:400) and biotinylated horse anti-mouse (Biozol, 1:2000, for 2 h, at RT. Following a washing step, FITC-avidin was applied (1:300, 1 h, RT), then DAPI staining performed (1:8000, 10 min, RT) and sections were cover slipped with Citifluor (Citifluor Ltd.). For image capture and analysis, a conventional light/fluorescent Olympus BX50 microscope (Olympus Optical, Hamburg, Germany) with a black and white Spot Insight camera (Diagnostic Instruments, Visitron systems, Puchheim, Germany) was used. By means of image editing software (MetaMorph 5.05) the individual images were combined into red/green/blue color figure plates and the counting tool was applied for the 4 areas as indicated; brightness and contrast were adjusted, and the images stored as TIFF files (Adobe Photoshop 5.05). All sections were processed the same way to enable comparison.

For analysis of neurogenesis markers, immunohistochemistry was conducted on 50 µm thick free-floating sections cut on a freezing sliding microtome. Every 8^th^ section was sampled for staining through the entire septo-temporal axis of the hippocampus.

The following pre-treatment were used prior to immunohistochemistry: for BrdU staining, sections were first incubated in 2 N HCl at 37 °C for 30 min to denature the DNA; for Ki67, antigen retrieval was performed with heated citrate buffer (pH = 6.0) in a steamer (95–100 °C) for 15 min, followed by a cool down at room temperature for 30 additional minutes; no pre-treatment was required for DCX staining.

For all markers, endogenous peroxidase was quenched in 3% H_2_O_2_ in PBS, followed by blocking (PBS/0.2% Triton x-100/2% normal serum from secondary antibodies host species) and overnight incubation in primary antibody solution diluted in blocking buffer at 4 °C (rat anti-BrdU 1:1000, Serotec; mouse anti-ki67 1:2000, Pharmingen; goat anti-DCX 1:1000, Santa Cruz Biotechnology). Sections were incubated for 1 hr at RT with biotinylated secondary antibodies at 1:200 (goat anti-rat, horse anti-mouse and horse anti-goat, respectively; Vector Laboratories), and the reaction was visualized with DAB after avidin-biotin peroxidase complex amplification (Vectastain, Vector Laboratories). Sections were then mounted on slides, left to dry overnight, dehydrated and coverslipped for stereological analysis.

Cell counting and density measurements were performed with an Olympus BX51 microscope under a ×40 objective lens and using Stereo Investigator software (MBF Bioscience). For each marker and in every 8^th^ section, all positive cells within the mounted thickness of the granule cell layer of the dentate gyrus were counted blind to the experimental group. For each section, counts were divided by the area of the dentate gyrus to yield cellular density. Data presented correspond to the average of 9 sections per animal.

### In vivo extracellular recording

In vivo single-unit extracellular recording electrophysiology experiments were conducted as previously^37^. Mice were anesthetized with chloral hydrate (400 mg kg^−1^ i.p., using a 2% solution) and placed in a stereotaxic frame (using the David Kopf mouse adaptor) with the skull positioned horizontally. To maintain a full anesthetic state in which there was no reaction to a tail or paw pinch, chloral hydrate supplements of 100 mg kg^−1^ were given as needed. Extracellular single-unit recordings were performed using single-barreled glass micropipettes pulled from 2 mm capillary glass (Stoelting, Wood Dale, IL, U.S.A.) on a PE-21 pipette puller (Narashige, Tokyo, Japan) and preloaded with fiberglass strands to promote capillary filling with 2 % Pontamine Sky Blue dye in 2 M NaCl for Dorsal Raphe Nuclei neuronal recordings. The micropipette tips were broken down to diameters of 1–3 μm to reach an electrode impedance of 2–6 MΩ.

For microinjection in the DRN, a cannula (33-gauge stainless steel tubing) was stereotaxically lowered using the following coordinates: AP: −3.42 mm from bregma; ML: ± 0.0 mm, DV: −1.97 mm from dura, 30° angle. For microinjections in the mPFC, a similar cannula was lowered at: AP: + 1.7 mm; ML: ± 2.0 mm; DV: −2.0 mm, 30° angle. Vehicle (ACSF: 2.5 mM KCl; 125 mM NaCl; 1.18 mM MgCl_2_; 1.26 mM CaCl_2_), TNFα (0.1 fg in 0.5 µl ACSF) or DN TNF (200 ng ml^−1^ in 0.5 ul) solutions were used to fill the cannula. The microinjection was made using a 5 μl Hamilton syringe that was connected to the injection cannula by a length of polyethylene (PE-10) tubing over a period of 60 s and the injection cannula was gently removed 2 min later.

The single-barreled glass micropipettes were positioned 0.5 to 1 mm posterior to the interaural line on the midline and lowered into the DRN, attained at a depth of between 2.5 and 3.5 mm from the brain surface. The DRN 5-HT neurons were then identified according to the following criteria: a slow (0.5–2.5 Hz) and regular firing rate with a long duration (0.8–1.2 ms) and positive action potentials. Single-unit activity was recorded as large-amplitude action potentials captured by a software window discriminator, amplified by an AC Differential MDA, 3 amplifier (BAK electronics, Umatilla, FL, U.S.A.), post-amplified and band-pass filtered by a Realistic 10 band frequency equalizer, digitized by a CED 1401 interface system (Cambridge Electronic Design, Cambridge, U.K.), processed online, and analyzed off-line using Spike2 software version 5.20 for Windows. The spontaneous single-spike activity of neurons was recorded for at least 2 min and 30 s immediately after detection to eliminate mechanical artifacts due to electrode displacement. The total number of spontaneously active 5-HT neurons and their firing rates were determined by monitoring their average discharge frequency for a minimum period of 1 min. The number of neurons recorded per electrode descent was also calculated, since the number of neurons recorded is a valid, indirect index of the percentage of neurons that are spontaneously discharging (active) during in vivo electrophysiological recordings^69^.

### Intracerebroventricular (ICV) TNFα injection

For ICV implantation, mice were anesthetized with chloral hydrate and placed in a stereotaxic frame with the skull positioned horizontally. Burr holes were drilled in the skull on the side over one of the lateral ventricle and a guide stainless steel cannula (26 gauge) was stereotaxically lowered 2.5 mm using the following coordinates: AP: −0.2 mm from bregma, L: ± 0.9 from the midline and V: −2.5 mm from cortical surface. The cannula was fixed into the skull using dental screws and cement. A vehicle (ACSF: 2.5 mM KCl; 125 mM NaCl; 1.18 mM MgCl_2_; 1.26 mM CaCl_2_) or recombinant mouse TNFα (R&D System) (0.1 fg in 5 µl ACSF) solution prefilled inner cannula (33-gauge stainless steel tubing) was inserted and driven 0.5 mm below the tip of the guide. The microinjection was made using a 5 μl Hamilton syringe that was connected to the injection cannula by a length of polyethylene (PE-10) tubing over a period of 60 s and the injection cannula was gently removed 2 min later.

### Whole cell recordings in PFC slices in vitro

*Eif4e* ki/ki and wild-type littermate mice (6–8-week-old) received intraperitoneal injections of ketamine (IP, 10 mg kg^‒1^) or saline 24 h before whole cell recording experiments. Serotonin creatine sulfate monohydrate (5-HT; 20, 50 or 100 µM; from Sigma) was freshly prepared in aCSF. Recombinant mouse TNF-α (R&D Systems) was dissolved in phosphate buffer containing 0.1% bovine serum albumin as stock solution (1000 × final concentration) and kept at ‒20 °C. The dominant-negative (DN) TNF, XPRO1595 (from Xencor), was freshly prepared in phosphate buffer. DN-TNF (200 ng ml^‒1^) and TNF-α (10 ng ml^‒1^) were freshly prepared in aCSF and slices were pre-incubated for 60 min before recording.

For brain slice preparation, *Eif4e* ki/ki and wild-type littermate mice (6 to 8-week-old) were anesthetized with isoflurane and the brain was removed and placed in ice-cold oxygenated (95% O_2_ / 5% CO_2_) cutting solution containing 87 mM NaCl, 2.5 mM KCl, 1.25 mM NaH_2_PO_4_, 7 mM MgSO_4_, 25 mM NaHCO_3_, 25 mM D-glucose, 75 mM sucrose, 1 mM ascorbic acid, 3 mM pyruvic acid and 0.5 mM CaCl_2_. Coronal slices (300 μm thickness) of medial prefrontal cortex were prepared using a Vibratome (Leica; VT1000S) and transferred to oxygenated artificial cerebrospinal fluid (aCSF) containing 124 mM NaCl, 5 mM KCl, 1.25 mM NaH_2_PO_4_, 2 mM MgSO_4_, 2 mM CaCl_2_, 26 mM NaHCO_3_ and 10 mM dextrose (pH = 7.3–7.4; 295-300 mOsmol) at room temperature. After a recovery period of at least 60 min, individual slices were transferred to a submersion recording chamber mounted on an upright microscope (Zeiss, Oberkochen, Germany) equipped with a long-range water immersion objective (40 ×) with Hoffmann optics (Modulation Optics, Greenvale, NY) and an infrared CCD camera, and perfused at 2.5 ml min^‒1^ with aCSF at 32 °C.

Recordings were obtained from layer V pyramidal cells under visual guidance using patch pipettes (3-5 MΩ) pulled from borosilicate glass capillaries (World Precision Instruments, Sarasota, USA) and filled with solution containing 120 mM CsMeSO_3_, 5 mM NaCl, 1 mM MgCl_2_, 10 mM di-Na-phosphocreatine, 10 mM HEPES, 2 mM ATP, 0.5 mM GTP, 2 mM QX314, 0.5 mM spermine and 0.3 % biocytin (pH 7.3; 285-295 mOsmol). Excitatory postsynaptic currents (EPSCs) were recorded in voltage-clamp mode using a Multiclamp 700 A amplifier (Molecular Devices) and digitized using Digidata 1440 A and pClamp10 software (Molecular Devices). Recordings were low-pass filtered at 2 kHz, digitized at 20 kHz and stored on a PC. Series resistance (15–25 MΩ) was regularly monitored during experiments and only cells with series resistance changes less than 20 % of initial value and stable holding current were included. Spontaneous EPSCs were recorded at −70 mV holding potential before, during, and after bath application of 5-HT (20 µM; 3 min application), and analyzed offline with Mini Analysis Version 6.0.3 software (Synaptosoft Inc., Decatur, GA, USA)^70^. The detection threshold of spontaneous EPSCs was set at twice baseline noise level and individual events were confirmed by visual inspection. Only cells that exhibited stable sEPSC frequency and amplitude over a 10 min control period were kept and only one cell was recorded per slice. After completion of recordings, slices were transferred to 4% paraformaldehyde (0.1 M phosphate buffer) and stored overnight at 4 °C. Slices were then processed using the Vectastain ABC kit (Vector Laboratories, Burlingame, CA, USA) and observed under a light microscope. Experimenter was blind to the mice genotype and codes were broken after data analysis.

### Statistical analyses

Data were expressed as mean ± S.E.M. and analyzed using GraphPad Prism 6 (GraphPad Software Inc.). Statistical results are detailed in Supplementary Table 2. When comparing two data sets, (Figs. 2d, i, 4c, d, 5d, i, 8f, h, k, n, Supplementary Figs. 1a, b, 3c, d, 6a–g) unpaired Student’s t-test were performed; when variance was significantly different between groups, Welch’s correction was used. One-way ANOVA was used for data in Fig. 3c, d. Data sets depicted in Figs. 1a–j, 3a, b, 4g, 5j, l, 6a–f, 7e, Supplementary Figs. 3a, 4f–h, 5a, 5c, 5e, 6h, and 7a–c were analyzed using two-way ANOVA, with genotype and gender (Fig. 1a, j, Supplementary Figs. 5a, c, e) or treatment (Figs. 3a, b, 4g, 5j, l, 6a–f, 7e, Supplementary Figs. 4f–h, 6h, 7a–c) as independent factors; when interaction was significant, Bonferroni pair-wise comparisons were used. Data from Figs. 2b, 4f, 7c, d, 8b, 8e, g, j, l and Supplementary Figs. 1c–f, 2a–c, 2f, g, 3a, b and 4b–e were analyzed using mixed-design two-way ANOVA with genotype (Fig. 2b, Supplementary Figs. 1c–f, 2a–c, 3a, b and 4b–e) or treatment (Figs. 4f, 7c, d, 8b, e, g, j, l, and Supplementary Figs. 2f, g) as independent factor, and pre- post- 5-HT (Figs. 2b, 4f, I, j, Supplementary Figs. 2a–c, 2f, g and 4b–e), time after treatment (Figs. 8b, d, f, i, k), testing session (Supplementary Figs. 1c–f) or drug dose (Supplementary Fig. 3a, b) as repeated measure; Bonferroni was used for pair-wise comparisons when interaction was significant. Values of p < 0.05 were considered significant.

### Data availability

The data that support the findings of this study are available from the corresponding author upon reasonable request.

## Electronic supplementary material

Supplementary Information

Peer Review File
