# Supplementary material for: Translational control of depression-like behavior via phosphorylation of eukaryotic translation initiation factor 4E
Source: Nat Commun. 2018 Jun 25;9:2459. doi: 10.1038/s41467-018-04883-5 (PMC6018502; doi:10.1038/s41467-018-04883-5)

Translational control of depression-like behavior via phosphorylation of eukaryotic translation initiation factor 4E

Argel Aguilar-Valles, Nabila Haji, Danilo De Gregorio, Edna Matta-Camacho, Mohammad J. Eslamizade, Jelena Popic, Vijendra Sharma, Ruifeng Cao, Christoph Rummel, Arnaud Tanti, Shane Wiebe, Nicolas Nunez, Stefano Comai, Robert Nadon, Giamal Luheshi, Naguib Mechawar, Gustavo Turecki, Jean-Claude Lacaille, Gabriella Gobbi and Nahum Sonenberg

Supplementary Information

Supplementary Figures

Supplementary Figure 1

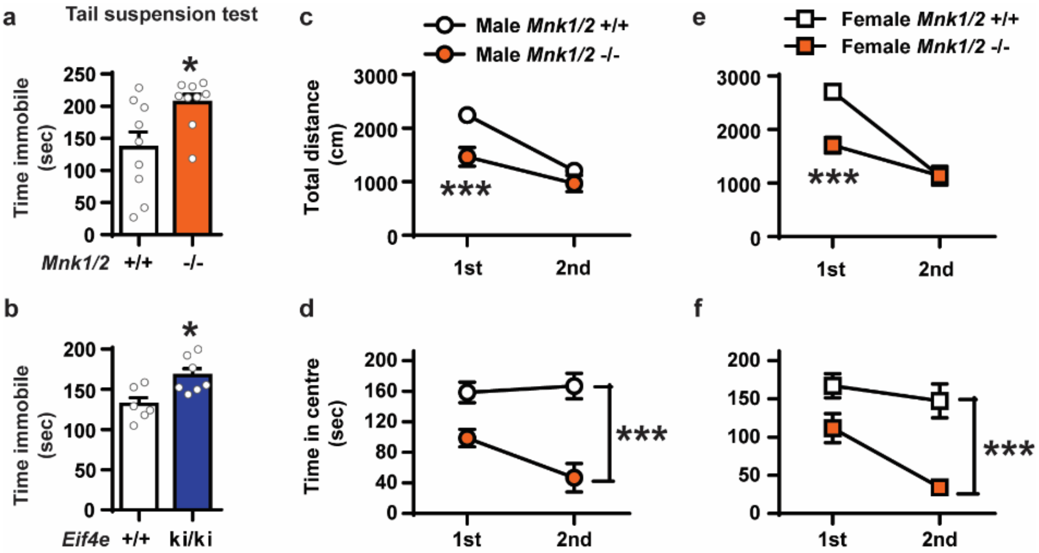

Supplementary Figure 1. Additional measures of despair-like and anxiety behaviors in *Eif4e* ki/ki and *Mnk1/2* -/- mice.

a, Tail suspension test (TST) was performed in male *Mnk1/2* -/- mice (n=9) and their wildtype littermates (n=9). **b,** TST was also performed in male *Eif4e* +/+ (n=6) and ki/ki mice (n=7). **c,** Male *Mnk1/2* +/+ and -/- mice locomotor activity in an open field (OF) was measured in two sessions 7 days apart from each other (data from 1st session is also presented in Fig. 1i) (n=13 *Mnk1/2* +/+, n=8 *Mnk1/2* -/-). **d,** Time of exploration of the OF center was recorded at the same time in the male mice (data from 1st session is also presented in Fig. 1g). **e,** Locomotor activity in the OF for female wildtype and *Mnk1/2* -/- mice was measured in two sessions 7 days apart (data from the 1st session is also presented in Fig. 1i) (n=7 *Mnk1/2* +/+, n=14 *Mnk1/2* -/-). **f,** Time of exploration of the OF center was recorded at the same time (data from 1st session is also presented in Fig. 1g). * p<0.05, *** p<0.001 vs. wildtype mice (see Supplementary Table 2 for detailed results of statistical tests).

Supplementary Figure 2

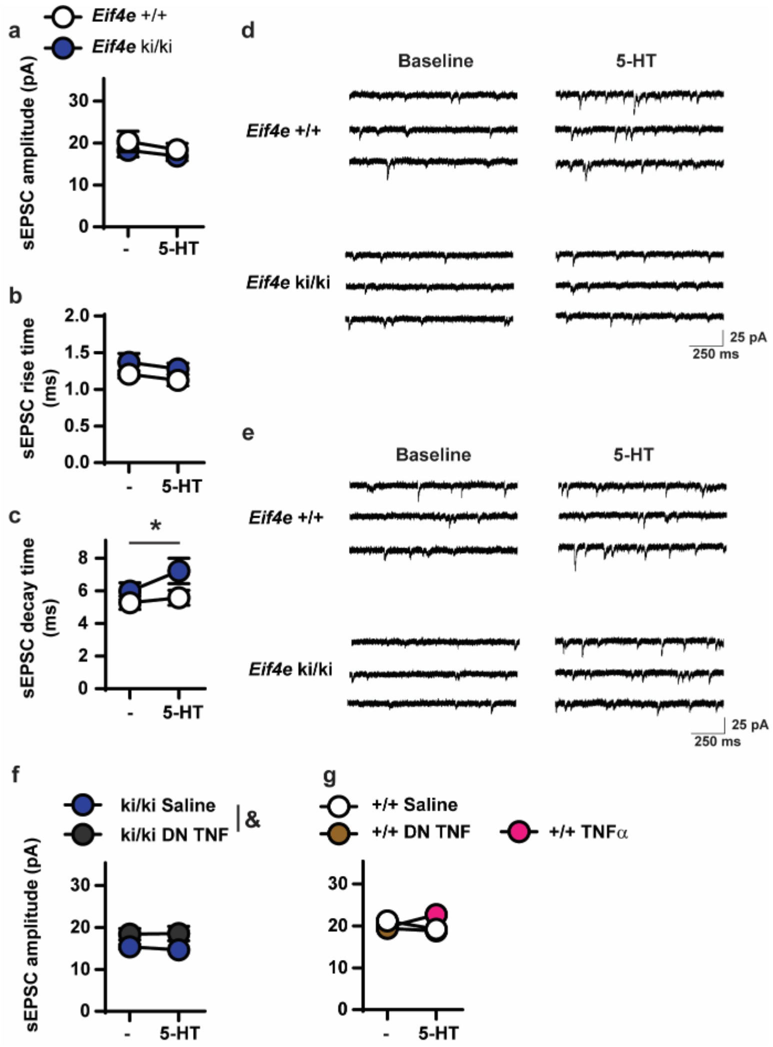

Supplementary Figure 2. Amplitude of spontaneous excitatory post-synaptic currents (sEPSCs) in layer V pyramidal neurons of mPFC remained unaltered across different experiments in Eif4e +/+ and ki/ki mice.

a, sEPSC amplitude in mPFC pyramidal neurons of *Eif4e* +/+ and ki/ki mice (from recordings in Fig. 1b, left panel). Synaptic currents were recorded before and after 5-HT (20 μM) bath application. Representative traces are shown in Figs. 1a (n). **b,** sEPSC rise time for recordings in **a** and Fig. 1b (left panel). **c,** sEPSC decay time for recordings in **a** and Fig. 1b (left panel). **d,e,** Representative whole-cell recordings of sEPSCs from layer V pyramidal neurons of mPFC of wildtype and *Eif4e* ki/ki mice. Synaptic currents were recorded before (baseline) and after 50 μM (**d**) or 100 μM (**e**) 5-HT treatment. Frequency data is presented in Fig. 1b (middle and right panel). **f,** Amplitude of sEPSCs of pyramidal neurons from *Eif4e* ki/ki mice in slices treated with either saline or DN TNF (200 ng/ml) and recorded pre- and post-5-HT (20 μM) application. Representative traces are shown in Fig. 3g. **g,** Amplitude of sEPSCs from wildtype mice in slices treated with either saline, DN TNF (200 ng/ml) or mouse recombinant TNFα (10 ng/ml). Representative traces are shown in Fig. 3h. *, $p<0.05$ 5-HT vs baseline (see Supplementary Table 2 for detailed results of statistical tests).

Supplementary Figure 3

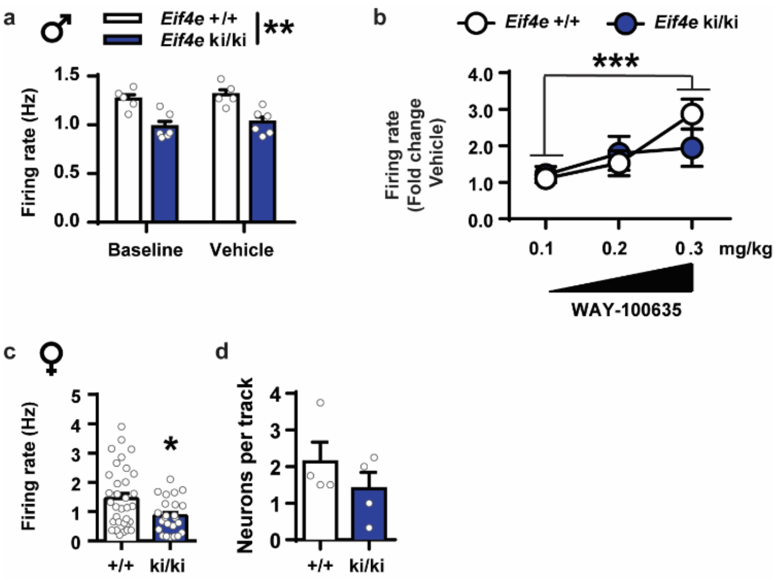

Supplementary Figure 3. Normal response to 5-HT$_{1A}$ autoreceptor inhibition and decrease 5-HT firing in female *Eif4e* ki/ki mice.

a, Firing rate of DR neurons in male wildtype and *Eif4e* ki/ki mice without treatment (baseline) or injected with vehicle (saline) (n=5 *Eif4e* +/+, n=6 *Eif4e* ki/ki; 1 recording per mouse). **b,** Firing rate of DR neurons in male wildtype and *Eif4e* ki/ki mice, following treatment with increasing doses of WAY-100635 (IP, 0.1-0.3 mg/kg), a 5HT$_{1A}$ antagonist. Data is expressed as change from vehicle condition depicted in panel **a**. **c,** 5-HT neuronal firing rate in female wildtype and *Eif4e* ki/ki mice. **d,** Number of 5-HT neurons per descent in the DR of female wildtype and *Eif4e* ki/ki mice. *, p<0.05, ** p<0.01, *** p<0.001 vs wildtype (see Supplementary Table 2 for detailed results of statistical tests).

Supplementary Figure 4

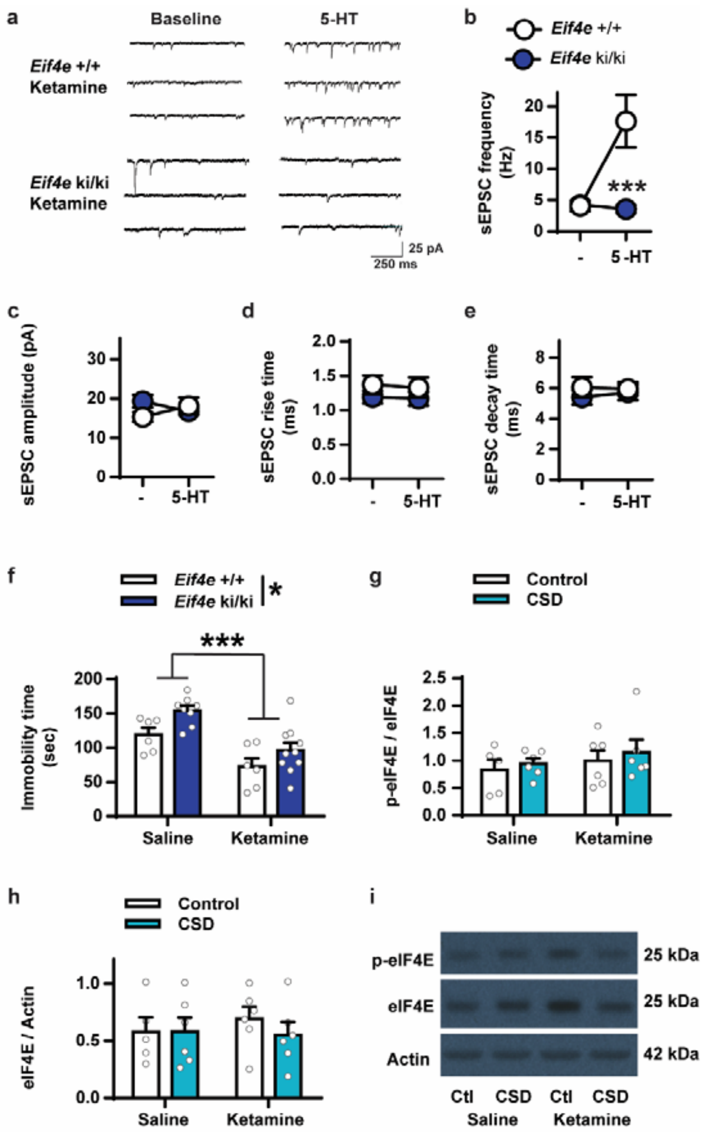

Supplementary Figure 4. Normal behavioral anti-depressant effect of ketamine in *Eif4e* ki/ki mice despite impaired serotonergic neurotransmission.

a, Representative sEPSCs following ketamine treatment (10 mg/kg, 24 h prior to recording) in *Eif4e* +/+ and ki/ki mice. **b,** Frequency of sEPSC in ketamine-treated wildtype or Eif4e ki/ki mice before and after 5-HT (20 μM) treatment (n=8 cells from 4 *Eif4e* +/+; n=13 cells from 5 *Eif4e* ki/ki). **c,** sEPSC amplitude in mPFC pyramidal neurons of *Eif4e* +/+ and ki/ki mice treated with ketamine (10 mg/kg) 24 h before recording. **d,** sEPSC rise time from recordings in panels **a-c**. **e,** sEPSC decay time of recordings in panels **a-c**. **f,** Immobility time in the FST 1 h after saline or ketamine treatment (IP, 10 mg/kg) in wildtype (Saline n=6; Ketamine n=6) or *Eif4e* ki/ki (Saline n=8; Ketamine n=10) mice. **g,** Phosphorylation levels of eIF4E in the mPFC/HPC of wildtype mice treated with saline or ketamine (1 h, IP, 10 mg/kg). Mice were either naïve or subjected to a chronic social defeat (CSD) protocol to induce depression-like phenotypes (n=5-6/group). **h**, Total levels of eIF4E in samples from **g**. **i,** Representative Western blots from mice treated with saline or ketamine. * p<0.05, *** p<0.001 vs. *Eif4e* +/+ or saline-treated groups.

Supplementary Figure 5

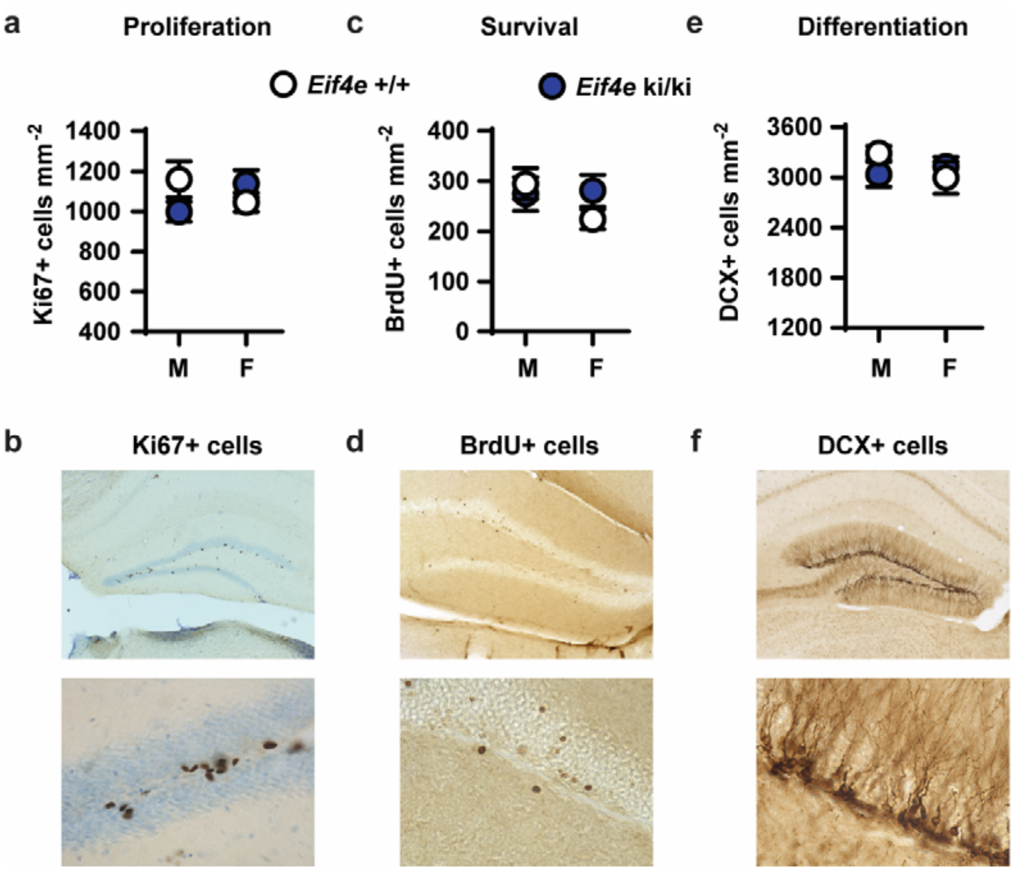

Supplementary Figure 5. Normal adult hippocampal neurogenesis in male and female *Eif4e* ki/ki mice.

a, Cell proliferation in adult dentate gyrus was determined by quantification of Ki67 positive cells in male and female wildtype and *Eif4e* ki/ki mice. **b,** Representative staining image for Ki67 + cells in the adult dentate gyrus. **c,** Survival of newborn cells in the adult dentate gyrus of male and female *Eif4e* +/+ and ki/ki mice was estimated by quantifying bromodeoxyuridine (BrdU) positive cells, 4 weeks after the last BrdU administration (IP). **d,** Representative staining image for BrdU + cells in the adult dentate gyrus. **e,** Differentiation of newborn cells into neurons in the adult dentate gyrus of male and female wildtype and *Eif4e* ki/ki mice was determined by quantification of doublecortin (DCX) positive cells. **f,** Representative staining of DCX positive cells in the adult dentate gyrus (see Supplementary Table 2 for group sizes and detailed results of statistical tests).

Supplementary Figure 6

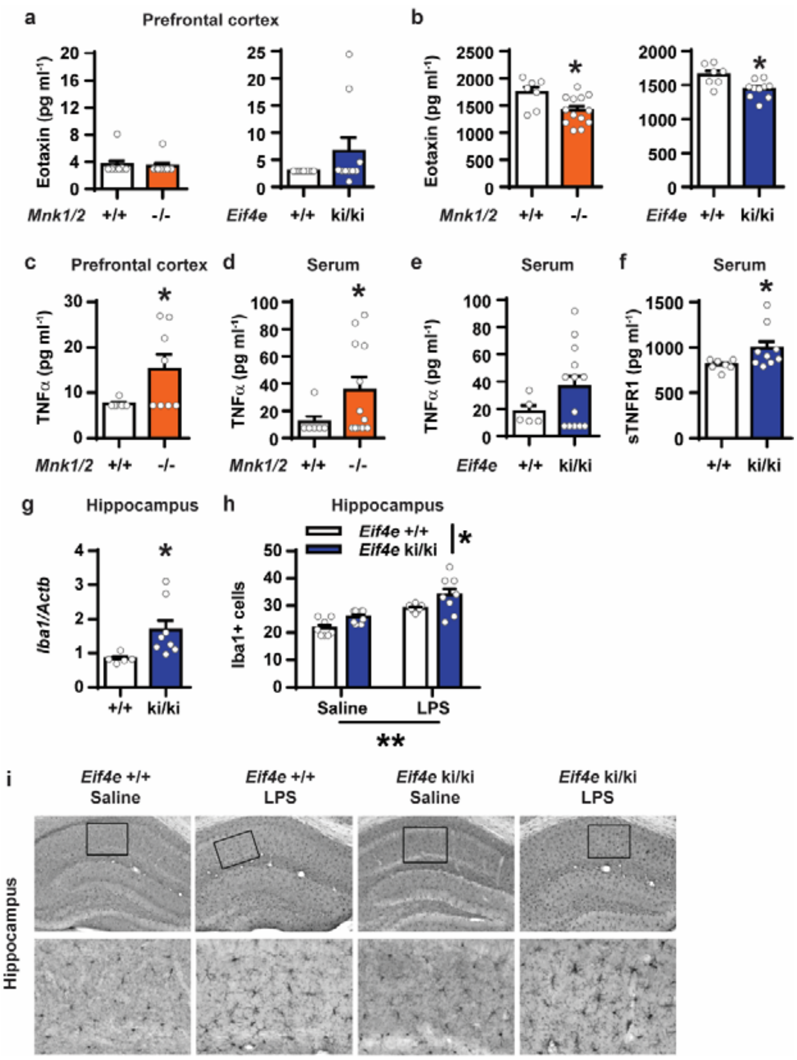

Supplementary Figure 6. Elevated TNFα and signs of neuroinflammation in *Mnk1/2* -/- and *Eif4e* ki/ki mice.

a, Eotaxin levels in the PFC of *Mnk1/2* -/- and *Eif4e* ki/ki mice and their respective littermate control mice (n=9 *Mnk1/2* +/+, n=9 *Mnk1/2* -/-; n=11 *Eif4e* +/+; n= 10 *Eif4e* ki/ki). **b,** Eotaxin was also measured in the peripheral blood of *Mnk1/2* -/- and *Eif4e* ki/ki mice (n=7 *Mnk1/2* +/+, n=13 *Mnk1/2* -/-; n=7 *Eif4e* +/+; n=10 *Eif4e* ki/ki). **c,** TNFα levels in the PFC of *Mnk1/2* -/- and wildtype mice (n=7 *Mnk1/2* +/+, n=8 *Mnk1/2* -/-). **d,** TNFα levels in the peripheral blood of *Mnk1/2* -/- and wildtype mice (n=6 *Mnk1/2* +/+, n=12 *Mnk1/2* -/-). **e,** TNFα levels in the peripheral blood of *Eif4e* ki/ki and wildtype mice (n=5 *Eif4e* +/+; n=13 *Eif4e* ki/ki). **f,** Soluble TNFα receptor 1 (sTNFR1) levels in the circulation of *Eif4e* ki/ki and wildtype mice (n=7 *Eif4e* +/+; n=9 *Eif4e* ki/ki).**g,** Steady-state mRNA levels of the microglial activation marker Iba1 (*Aif1*) in the hippocampus of wildtype and *Eif4e* ki/ki mice (n=5 *Eif4e* +/+; n=8 *Eif4e* ki/ki). **h,** Number of Iba1 positive cells per 0.01 mm^2 of hippocampus from wildtype and *Eif4e* ki/ki mice treated with saline or LPS (IP, 2.5 mg/kg, IP) 48 h before tissue collection (n=4/group). **h,** Representative images of immune-staining for Iba1 in the hippocampus of wildtype or *Eif4e* ki/ki mice. * $p<0.05$, ** $p<0.01$ vs wildtype (see Supplementary Table 2 for detailed results of statistical tests).

Supplementary Figure 7

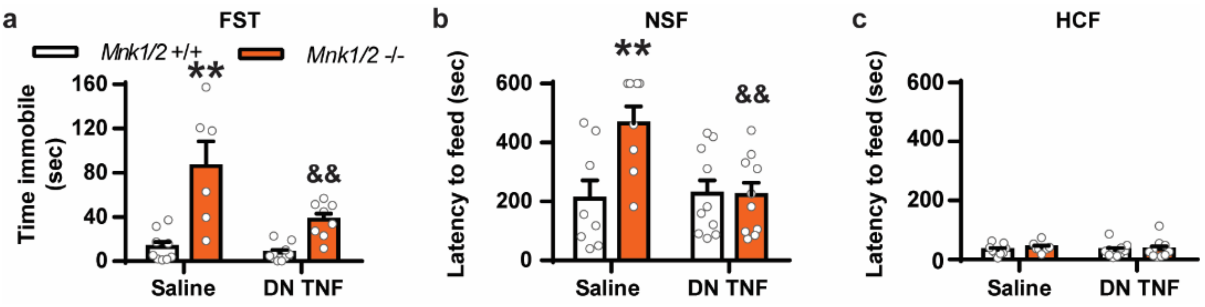

Supplementary Figure 7. Inhibition of TNFα reverses depression-like behavior in *Mnk1/2* -/- mice.

a, Effect of chronic administration of saline or dominant negative TNF (DN TNF, ICV) in wildtype and *Mnk1/*2 -/- mice in the FST (n=9 *Mnk1/2* +/+ Saline, n=10 *Mnk1/2* +/+ DN TNF, n=6 *Mnk1/2* -/- Saline, n=8 *Mnk1/2* -/- DN TNF). **b,** These mice were also evaluated in the novelty suppressed feeding (NSF) task (n=8 *Mnk1/2* +/+ Saline, n=10 *Mnk1/2* +/+ DN TNF, n=8 *Mnk1/2* -/- Saline, n=10 *Mnk1/2* -/- DN TNF). **c,** Home-cage feeding (HCF) was also determined in wildtype and *Mnk1/2* -/- mice. ** p<0.01 vs. wildtype; && p<0.01 vs. *Mnk1/2* -/- treated with saline (see Supplementary Table 2 for detailed results of statistical tests).

Supplementary Figure 8

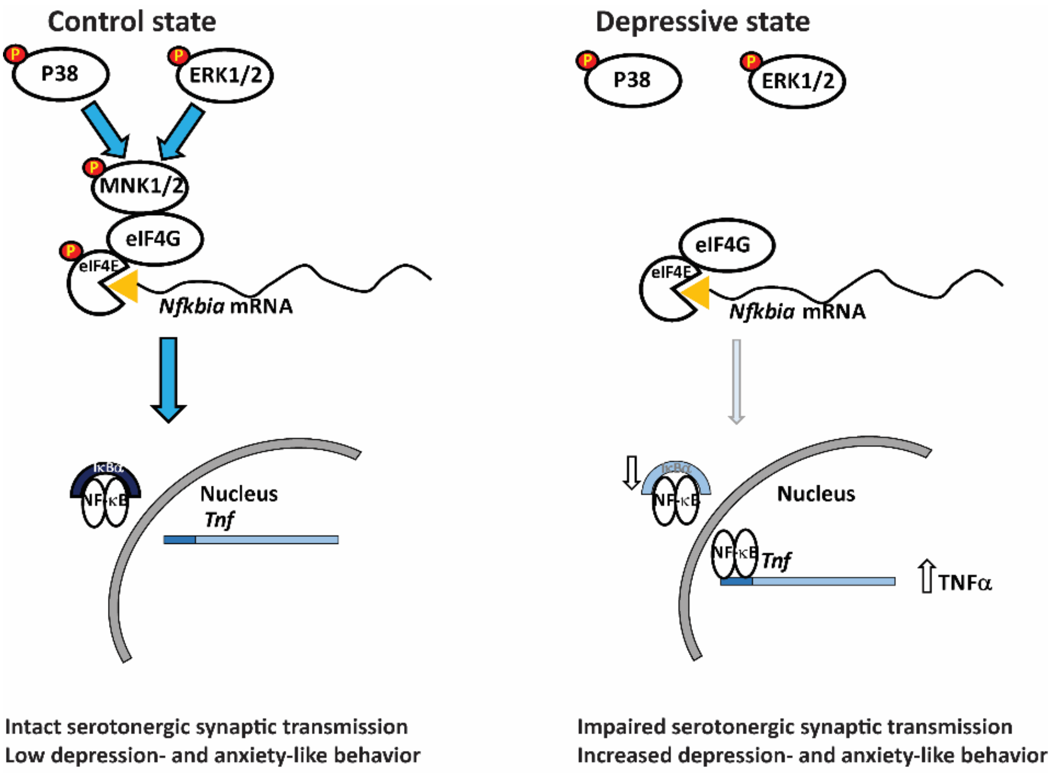

Supplementary Figure 8. Contribution of the eIF4E phosphorylation pathways to depression pathophysiology.

In a control state, eIF4E phosphorylation stimulates *Nfkbia* translation, which contributes to the baseline repression of the NF-κB complexes and thus keeps the levels of transcription of *Tnf* inhibited. In the absence of Mnk1/2 or by mutating the unique phosphorylation site in eIF4E, translation of *Nfkbia* is decreased, leading to increased activity of the NF-κB complex and increased levels of *Tnf* translation. Increased TNF*α* produces serotonergic dysfunction and depression- and anxiety-like behaviors.

Supplementary Tables

Supplementary Table 1. Prefrontal cortex levels of cytokines

Cytokine	*Mnk1/2* +/+	*Mnk1/2* -/-	*Eif4e* +/+	*Eif4e* ki/ki
	n=9	n=9	n=8	n=8
IL-1a	100 ± 8.36	88.92 ± 3.34	100 ± 6.39	114.84 ± 4.89
IL-1b	100 ± 3.43	96.53 ± 3.09	100 ± 5.46	110.20 ± 3.16
IL-2	100 ± 12.75	78.50 ± 7.60	100 ± 14.27	110.20 ± 8.88
IL-5	100 ± 6.90	89.47 ± 7.59	100 ± 4.38	104.77 ± 5.32
IL-6	100 ± 3.16	102.13 ± 14.77	100 ± 8.77	102.74 ± 9.16
IL-9	100 ± 15.54	131.13 ± 13.03	100 ± 15.81	102.61 ± 14.60
IL-10	100 ± 6.83	98.65 ± 4.75	100 ± 7.88	99.04 ± 6.83
IL-12 (p40)	100 ± 5.55	121.03 ± 15.18	100 ± 21.94	93.06 ± 5.95
IL-12 (p70)	100 ± 10.13	119.94 ± 8.58	100 ± 16.34	113.85 ± 12.35
IL-13	100 ± 5.32	90.26 ± 3.69	100 ± 7.46	116.29 ± 10.79
IL-17	100 ± 6.32	70.31 ± 3.11	100 ± 8.94	95.25 ± 10.02
Eotaxin	100 ± 10.63	124.09 ± 7.58	100 ± 13.76	127.90 ± 7.49
GM-CSF	100 ± 7.03	101.87 ± 3.16	100 ± 7.16	106.70 ± 4.79
IFN-g	100 ± 9.17	116.14 ± 9.23	100 ± 13.47	109.05 ± 7.58
KC	100 ± 3.70	96.13 ± 4.98	100 ± 9.35	102.50 ± 4.69
MCP-1	100 ± 5.14	106.98 ± 4.98	100 ± 7.37	104.71 ± 4.51
MIP-1a	100 ± 17.84	84.60 ± 7.13	100 ± 12.91	93.49 ± 4.68
MIP-1b	100 ± 3.25	91.49 ± 2.76	100 ± 5.67	100.36 ± 3.43
RANTES	100 ± 8.04	125.25 ± 38.97	100 ± 26.94	73.38 ± 6.94
TNFa	100 ± 20.36	1783.62 ± 885.43	100 ± 31.88	460.70 ± 308.25
IL-3	n.d.	n.d.	n.d.	n.d.
IL-4	n.d.	n.d.	n.d.	n.d.
G-CSF	n.d.	n.d.	n.d.	n.d.

Data is expressed as % of respective wildtype group. Data is average +/- S.E.M. n.d.: non detectable

Supplementary Table 2. Statistical analyses

Figure	Panel	Test	Group size	Statistic	P value	Pair-wise comparisons	Statistic
**Fig. 1**	**a**	2-way ANOVA (Gender X Genotype)	Males: Mnk1/2 +/+ = 8 Mnk1/2 -/- = 9	Main effect of genotype: F(1, 25) = 13.37	0.0012	N/A	
			Females: Mnk1/2 +/+ = 3 Mnk1/2 -/- = 9	Main effect of gender: F(1, 25) = 0.3217	0.5757	N/A	
				Gender X Genotype Interaction: F(1, 25) = 1.509	0.2307	N/A	
	**b**	2-way ANOVA (Gender X Genotype)	Males: Eif4e +/+ = 19 Eif4e ki/ki = 19	Main effect of genotype: F(1, 49) = 11.85	0.0012	N/A	
			Females: Eif4e +/+ = 7 Eif4e ki/ki = 8	Main effect of gender: F(1, 49) = 1.690	0.1996	N/A	
				Gender X Genotype Interaction: F(1, 49) = 0.2206	0.6407	N/A	
	**c**	2-way ANOVA (Gender X Genotype)	Males: Mnk1/2 +/+ = 8 Mnk1/2 -/- = 9	Main effect of genotype: F(1, 33) = 1.394	0.2461	N/A	
			Females: Mnk1/2 +/+ = 10 Mnk1/2 -/- = 10	Main effect of gender: F(1, 33) = 3.970	0.0547	N/A	

				Gender X Genotype Interaction: F(1, 19) = 18.45	0.0163	Bonferroni	Males Mnk1/2 +/+ vs -/- t(33)=3.26, p<0.01
						Bonferroni	Females Mnk1/2 +/+ vs -/- t(33)=1.80, p>0.05
	**d**	2-way ANOVA (Gender X Genotype)	Males: Eif4e +/+ = 9 Eif4e ki/ki = 9	Main effect of genotype: F(1, 31) = 12.66	0.0012	N/A	
			Females: Eif4e +/+ = 8 Eif4e ki/ki = 9	Main effect of gender: F(1, 31) = 21.20	< 0.0001	N/A	
				Gender X Genotype Interaction: F(1, 31) = 0.1191	0.7323	N/A	
	**e**	2-way ANOVA (Gender X Genotype)	Males: Mnk1/2 +/+ = 8 Mnk1/2 -/- = 9	Main effect of genotype: F(1, 33) = 1.903	0.177	N/A	
			Females: Mnk1/2 +/+ = 10 Mnk1/2 -/- = 10	Main effect of gender: F(1, 33) = 0.1129	0.739	N/A	
				Gender X Genotype Interaction: F(1, 33) = 4.54	0.0407	Bonferroni	Males Mnk1/2 +/+ vs -/- t(33)=0.51, p>0.05
						Bonferroni	Females Mnk1/2 +/+ vs -/- t(33)=2.59, p<0.05
	**f**	2-way ANOVA (Gender X Genotype)	Males: Eif4e +/+ = 9 Eif4e ki/ki = 9	Main effect of genotype: F(1, 31) = 0.3155	0.5784	N/A	

			Females:<br>Eif4e +/+ = 8<br>Eif4e ki/ki = 9	Main effect of gender: F(1, 31) = 1.345	0.255	N/A
				Gender X Genotype Interaction: F(1, 31) = 0.4449	0.5097	N/A
	**g**	2-way ANOVA (Gender X Genotype)	Males:<br>Mnk1/2 +/+ = 12 Mnk1/2 -/- = 8	Main effect of genotype: F(1, 36) = 20.01	< 0.0001	N/A
			Females:<br>Mnk1/2 +/+ = 7<br>Mnk1/2 -/- = 13	Main effect of gender: F(1, 36) = 0.3780	0.5425	N/A
				Gender X Genotype Interaction: F(1, 36) = 0.003936	0.9503	N/A
	**h**	2-way ANOVA (Gender X Genotype)	Males:<br>Eif4e +/+ = 17<br>Eif4e ki/ki = 18	Main effect of genotype: F(1, 61) = 20.72	< 0.0001	N/A
			Females:<br>Eif4e +/+ = 11<br>Eif4e ki/ki = 19	Main effect of gender: F(1, 61) = 2.174	0.1455	N/A
				Gender X Genotype Interaction: F(1, 61) = 0.2857	0.5949	N/A
	**i**	2-way ANOVA (Gender X Genotype)	Males:<br>Mnk1/2 +/+ = 12 Mnk1/2 -/- = 8	Main effect of genotype: F(1, 36) = 30.99	< 0.0001	N/A

			Females:<br>Mnk1/2 +/+ = 7<br>Mnk1/2 -/- = 13	Main effect of gender: F(1, 36) = 6.503	0.0152	N/A	
				Gender X Genotype Interaction: F(1, 36) = 0.7873	0.3808	N/A	
	j	2-way ANOVA (Gender X Genotype)	Males:<br>Eif4e +/+ = 17<br>Eif4e ki/ki = 18	Main effect of genotype: F(1, 61) = 1.317	0.2557	N/A	
			Females:<br>Eif4e +/+ = 11<br>Eif4e ki/ki = 19	Main effect of gender: F(1, 61) = 1.739	0.1923	N/A	
				Gender X Genotype Interaction: F(1, 61) = 0.01223	0.9123	N/A	
**Fig. 2**	**b**	20 µM 5-HT doseRepeated measures 2-way ANOVA (5-HT exposure X Genotype)	Eif4e +/+ = 7 cells<br>Eif4e ki/ki = 8 cells	Main effect of genotype: F(1, 13) = 2.110	0.1701		
				Main effect of 5-HT exposure: F(1, 13) = 5.899	0.0304		
				5-HT exposure X Genotype Interaction: F(1, 13) = 6.962	0.0205	Bonferroni	Baseline +/+ vs ki/ki t(26)=0.05899, p>0.05
						Bonferroni	5-HT +/+ vs ki/ki t(26)=2.576, p<0.05

	50 μM 5-HT doseRepeated measures 2-way ANOVA (5-HT exposure X Genotype)	Eif4e +/+ = 4 cells<br>Eif4e ki/ki = 5 cells	Main effect of genotype: F(1, 7) = 5.883	0.0457		
			Main effect of 5-HT exposure: F(1, 7) = 16.05	0.0051		
			5-HT exposure X Genotype Interaction: F(1, 7) = 22.22	0.0022	Bonferroni	Baseline +/+ vs ki/ki t(14)=0.133, p>0.05
					Bonferroni	5-HT +/+ vs ki/ki t(14)=4.235, p<0.01
	100 μM 5-HT doseRepeated measures 2-way ANOVA (5-HT exposure X Genotype)	Eif4e +/+ = 5 cells<br>Eif4e ki/ki = 6 cells	Main effect of genotype: F(1, 9) = 0.2451	0.6324		
			Main effect of 5-HT exposure: F(1, 9) = 13.93	0.0047		
			5-HT exposure X Genotype Interaction: F(1, 9) = 0.0329	0.86		

	**d**	Unpaired t-test	Eif4e +/+ = 56 cells<br>Eif4e ki/ki = 43 cells	t(97) = 5.533	< 0.0001	N/A
	**e**	Unpaired t-test	Eif4e +/+ = 8<br>Eif4e ki/ki = 7	t(13) = 1.451	0.1705	N/A
	**f**	Unpaired t-test	Eif4e +/+ = 27 cells<br>Eif4e ki/ki = 16 cells	t(41) = 0.7522	0.4562	N/A
	**g**	Unpaired t-test	Eif4e +/+ = 6<br>Eif4e ki/ki = 5	t(9) = 1.325	0.2179	N/A
	**h**	DRD Unpaired t-test	Eif4e +/+ = 4<br>Eif4e ki/ki = 5	t(7) = 1.076	0.3174	N/A
		DRL Unpaired t-test		t(7) = 0.0973	0.9252	N/A
		PDR Unpaired t-test		t(7) = 0.3572	0.7315	N/A
		DRV Unpaired t-test		t(7) = 2.080	0.0761	N/A
		DRD Unpaired t-test	Eif4e +/+ = 4<br>Eif4e ki/ki = 5	t(7) = 1.779	0.1185	N/A
		DRL Unpaired t-test		t(7) = 1.773	0.1196	N/A
		PDR Unpaired t-test		t(7) = 2.463	0.0437	N/A
		DRV Unpaired t-test		t(7) = 2.657	0.0326	N/A
**Fig. 3**	**a**	2-way ANOVA (Treatment X Genotype)	S Mnk1/2 +/+ = 7 F Mnk1/2 +/+ = 10	Main effect of genotype: F(1, 32) = 25.47	< 0.0001	N/A

			S Mnk1/2 -/- = 9<br>F Mnk1/2 -/- = 10	Main effect of treatment: F(1, 32) = 53.10	< 0.0001	N/A
				Treatment X Genotype Interaction: F(1, 32) = 2.795	0.1043	N/A
	**b**	2-way ANOVA (Treatment X Genotype)	S Eif4e +/+ = 9<br>F Eif4e +/+ = 6	Main effect of genotype: F(1, 28) = 23.46	< 0.0001	N/A
			S Eif4e ki/ki = 8<br>F Eif4e ki/ki = 9	Main effect of treatment: F(1, 28) = 7.860	0.0091	N/A
				Treatment X Genotype Interaction: F(1, 28) = 1.191	0.2844	N/A
	**c**	1-way ANOVA	Sal. = 6<br>Fluox. = 6<br>Chr. Fluox. = 6<br>Rep. Cital. = 6	F(3, 20) = 0.6547	0.5894	N/A
	**d**	1-way ANOVA	Sal. = 6<br>Fluox. = 6<br>Chr. Fluox. = 6<br>Rep. Cital. = 6	F(3, 20) = 0.9680	0.4273	N/A
**Fig. 4**	**c**	p-eIF4E/eIF4E Unpaired t-test	Vehicle = 7<br>Cercosporamide (20 mg/kg)= 7	t(12) = 2.994	0.0112	N/A
		eIF4E/GAPDH Unpaired t-test	Vehicle = 7<br>Cercosporamide (20 mg/kg)= 7	t(12) = 0.0840	0.9344	N/A

	**d**	Unpaired t-test	Vehicle = 8<br>Cercosporamide (20 mg/kg)= 7	t(13) = 2.645	0.0202	N/A	
	**f**	Eif4e +/+ Repeated measures 2-way ANOVA (5-HT exposure X Treatment)	Eif4e +/+ 0 mg/kg = 5 cells<br>Eif4e +/+ 20 mg/kg = 5 cells	Main effect of Treatment: F(1, 8) = 8.541	0.0192	N/A	
				Main effect of 5-HT exposure: F(1, 8) = 4.027	0.0797	N/A	
				5-HT exposure X Treatement Interaction: F(1, 8) = 7.093	0.0287	Bonferroni	Baseline 0 vs 20 mg/kg t(16)=1.157, p>0.05
						Bonferroni	5-HT 0 vs 20 mg/kg t(16)=3.872, p<0.01
		Eif4e ki/ki Repeated measures 2-way ANOVA (5-HT exposure X Treatment)	Eif4e ki/ki 0 mg/kg = 7 cells<br>Eif4e ki/ki 20 mg/kg = 5 cells	Main effect of Treatment: F(1, 10) = 1.913	0.1967	N/A	
				Main effect of 5-HT exposure: F(1, 10) = 0.3711	0.556	N/A	
				5-HT exposure X Genotype Interaction: F(1, 10)	0.794	N/A	

				= 0.072			
	g	Firing rate data<br>2-way ANOVA (Treatment X Genotype)	0 mg/kg Eif4e +/+ = 33<br>20 mg/kg Eif4e +/+ = 22	Main effect of genotype: F(1, 91) = 10.96	0.0013	Planed pair-wise comparisons with Bonferroni correction	0 mg/kg Eif4e +/+ vs Eif4e ki/ki t(91) = 3.825, p<0.01
			0 mg/kg Eif4e ki/ki = 22<br>20 mg/kg Eif4e ki/ki = 18	Main effect of treatment: F(1, 91) = 6.907	0.0101		0 mg/kg Eif4e +/+ vs 20 mg/kg Eif4e +/+ t(91) = 3.304, p<0.01
				Treatment X Genotype Interaction: F(1, 91) = 2.883	0.0929		0 mg/kg Eif4e ki/ki vs 20 mg/kg Eif4e ki/ki t(91) = 0.6153, p>0.05
		Neurons per track<br>2-way ANOVA (Treatment X Genotype)	0 mg/kg Eif4e +/+ = 6<br>20 mg/kg Eif4e +/+ = 10	Main effect of genotype: F(1, 21) = 0.009	0.9254	N/A	
			0 mg/kg Eif4e ki/ki = 4<br>20 mg/kg Eif4e ki/ki = 5	Main effect of treatment: F(1, 21) = 3.087	0.0935	N/A	
				Treatment X Genotype Interaction: F(1, 21) = 0.018	0.8935	N/A	
**Fig. 5**	**d**	Unpaired t-test	Eif4e +/+ = 3<br>Eif4e ki/ki = 3	t(4) = 0.2907	0.7857	N/A	

	**e**	Unpaired t-test	Eif4e +/+ = 6<br>Eif4e ki/ki = 6	t(10) = 2.679	0.0231	N/A
	**f**	Unpaired t-test	Eif4e +/+ = 7<br>Eif4e ki/ki = 8	t(13) = 2.202	0.0463	N/A
	**g**	Unpaired t-test with Welch's correction	Eif4e +/+ = 9<br>Eif4e ki/ki = 10	t(9.183) = 3.222	0.0102	N/A
	**h**	Unpaired t-test with Welch's correction	Eif4e +/+ = 10<br>Eif4e ki/ki = 12	t(12.92) = 2.227	0.0443	N/A
	**i**	Unpaired t-test	Eif4e +/+ = 6<br>Eif4e ki/ki = 5	t(9) = 2.973	0.0156	N/A
	**j**	2-way ANOVA (Treatment X Genotype)	S Eif4e +/+ = 3<br>L Eif4e +/+ = 3	Main effect of genotype: F(1, 8) = 6.4783	0.0344	N/A
			S Eif4e ki/ki = 3<br>L Eif4e ki/ki = 3	Main effect of treatment: F(1, 8) = 40.72	0.0002	N/A
				Treatment X Genotype Interaction: F(1, 8) = 2.516	0.1514	N/A
	**l**	2-way ANOVA (Treatment X Genotype)	S Eif4e +/+ = 6<br>L Eif4e +/+ = 3	Main effect of genotype: F(1, 15) = 7.299	0.0164	N/A
			S Eif4e ki/ki = 6<br>L Eif4e ki/ki = 4	Main effect of treatment: F(1, 15) = 25.09	0.0002	N/A

				Treatment X Genotype Interaction: F(1, 15) = 4.624	0.0482	Bonferroni	S Eif4e ki/ki vs L Eif4e ki/ki t(15) = 5.309, p<0.001
						Bonferroni	L Eif4e +/+ vs L Eif4e ki/ki t(15) = 3.041, p<0.05
**Fig. 6**	**a**	2-way ANOVA (Treatment X Genotype)	S Eif4e +/+ = 11 DN Eif4e +/+ = 7	Main effect of genotype: F(1, 32) = 3.193	0.0834	N/A	
			S Eif4e ki/ki = 10 DN Eif4e ki/ki = 8	Main effect of treatment: F(1, 32) = 5.703	0.023	N/A	
				Treatment X Genotype Interaction: F(1, 32) = 6.657	0.0147	Bonferroni	S Eif4e +/+ vs S Eif4e ki/ki t(32) = 3.385, p<0.01
						Bonferroni	S Eif4e ki/ki vs DN Eif4e ki/ki t(32) = 3.574, p<0.01
	**b**	2-way ANOVA (Treatment X Genotype)	S Eif4e +/+ = 10 DN Eif4e +/+ = 8	Main effect of genotype: F(1, 30) = 2.394	0.1323	N/A	
			S Eif4e ki/ki = 8 DN Eif4e ki/ki = 8	Main effect of treatment: F(1, 30) = 1.661	0.2073	N/A	
				Treatment X Genotype Interaction: F(1, 30) = 4.841	0.0356	Bonferroni	S Eif4e +/+ vs S Eif4e ki/ki t(30) = 2.722, p<0.05
						Bonferroni	S Eif4e ki/ki vs DN Eif4e ki/ki t(30) = 2.405, p<0.05

	**d**	2-way ANOVA (Treatment X Genotype)	S Eif4e +/+ = 7 DN Eif4e +/+ = 8	Main effect of genotype: F(1, 27) = 6.316	0.0182	N/A	
			S Eif4e ki/ki = 8 DN Eif4e ki/ki = 8	Main effect of treatment: F(1, 27) = 3.407	0.0759	N/A	
				Treatment X Genotype Interaction: F(1, 27) = 6.791	0.0147	Bonferroni	S Eif4e +/+ vs S Eif4e ki/ki t(27) = 3.559, p<0.01
						Bonferroni	S Eif4e ki/ki vs DN Eif4e ki/ki t(27) = 3.204, p<0.05
	**e**	2-way ANOVA (Treatment X Genotype)	S Eif4e +/+ = 7 DN Eif4e +/+ = 10	Main effect of genotype: F(1, 32) = 7.679	0.0092	N/A	
			S Eif4e ki/ki = 10 DN Eif4e ki/ki = 9	Main effect of treatment: F(1, 32) = 0.902	0.3495	N/A	
				Treatment X Genotype Interaction: F(1, 32) = 4.364	0.0447	Bonferroni	S Eif4e +/+ vs S Eif4e ki/ki t(32) = 3.322, p<0.05
**Fig. 7**	**c**	Males Repeated measures 2-way ANOVA (5-HT exposure X Treatment)	S Eif4e +/+ = 10 DN Eif4e +/+ = 8 TNFa Eif4e +/+ = 9	Main effect of 5-HT exposure: F(1, 24) = 44.10	P < 0.0001	N/A	
				Main effect of treatment: F(2, 24) = 13.48	P < 0.0001	N/A	

				5-HT exposure X Treatment inteaction: F(2, 24) = 7.604	P = 0.0028	Bonferroni	Eif4e +/+ Saline: Baseline vs 5-HT t(24) = 6.411, p<0.0001
						Bonferroni	Eif4e +/+ DN TNF: Baseline vs 5-HT t(24) = 4.374, p<0.001
		Females Repeated measures 2-way ANOVA (5-HT exposure X Treatment)	S Eif4e +/+ = 9 DN Eif4e +/+ = 7 TNFa Eif4e +/+ = 6	Main effect of 5-HT exposure: F(1, 19) = 15.56	P = 0.0009	N/A	
				Main effect of treatment: F(2, 19) = 8.662	P = 0.0021	N/A	
				5-HT exposure X Treatment inteaction: F(2, 19) = 5.224	P = 0.0156	Bonferroni	Eif4e +/+ Saline: Baseline vs 5-HT t(19) = 4.130, p<0.01
						Bonferroni	Eif4e +/+ DN TNF: Baseline vs 5-HT t(19) = 3.597, p<0.01
	**d**	Males Repeated measures 2-way ANOVA (5-HT exposure X Treatment)	S Eif4e ki/ki = 11 DN Eif4e ki/ki = 8	Main effect of 5-HT exposure: F(1, 17) = 19.96	0.0003	N/A	

				Main effect of treatment: F(1, 17) = 5.677	0.0291	N/A	
				5-HT exposure X Treatment Interaction: F(1, 17) = 16.59	0.0008	Bonferroni	Eif4e ki/ki DN TNF: Baseline vs 5-HT t(34) = 3.649, p<0.01
		Females Repeated measures 2-way ANOVA (5-HT exposure X Treatment)	S Eif4e ki/ki = 9 DN Eif4e ki/ki = 9	Main effect of 5-HT exposure: F(1, 16) = 7.442	0.0149	N/A	
				Main effect of treatment: F(1, 16) = 1.673	0.2143	N/A	
				5-HT exposure X Treatment Interaction: F(1, 16) = 12.65	0.0026	Bonferroni	Eif4e ki/ki DN TNF: Baseline vs 5-HT t(16) = 4.444, p<0.001
	**e**	2-way ANOVA (Treatment X Genotype)	S Eif4e +/+ = 33 DN Eif4e +/+ = 31	Main effect of genptype: F(1, 109) = 16.17	0.0001	N/A	
			S Eif4e ki/ki = 16 DN Eif4e ki/ki = 33	Main effect of treatment: F(1, 109) = 5.814	0.0176	N/A	
				Treatment X Genotype Interaction: F(1, 109) = 3.994	0.0482	Bonferroni	S Eif4e +/+ vs S Eif4e ki/ki t(109) = 3.895, p<0.001

						Bonferroni	Eif4e ki/ki S vs DN t(109) = 2.853, p<0.05
**Figure 8**	**b**	Repeated measures 2-way ANOVA (Time X Treatment)	S WT = 4<br>TNF WT = 6	Main effect of time: F(3, 24) = 28.96	< 0.0001	N/A	
				Main effect of treatment: F(1, 8) = 8.742	0.0182	N/A	
				Time X Treatment Interaction: F(3, 24) = 23.11	< 0.0001	Bonferroni	10 min S vs TNF t(32) = 4.068, p<0.01
						Bonferroni	15 min S vs TNF t(32) = 5.635, p<0.001
	**e**	Repeated measures 2-way ANOVA (Time X Treatment)	S WT = 6<br>TNFalpha WT = 6	Main effect of time: F(4, 50) = 2.794	0.0359	N/A	
				Main effect of treatment: F(1, 50) = 26.76	< 0.0001	N/A	
				Time X Treatment Interaction: F(4, 50) = 3.043	0.0254	Bonferroni	600 sec S vs TNF t(50) = 4.040, p<0.001
						Bonferroni	800 sec S vs TNF t(50) = 4.005, p<0.01
	**f**	Unpaired t-test	S WT = 6<br>TNFalpha WT = 7	t(11) = 3.432	0.0056	N/A	

	**j**	Repeated measures 2-way ANOVA (Time X Treatment)	S WT = 7 TNFalpha WT = 6	Main effect of time: F(4, 55) = 18.42	< 0.0001	N/A	
				Main effect of treatment: F(1, 55) = 173.1	< 0.0001	N/A	
				Time X Treatment Interaction: F(4, 55) = 16.41	< 0.0001	Bonferroni	200 sec S vs TNF t(55) = 3.462, p<0.01
						Bonferroni	400 sec S vs TNF t(55) = 7.466, p<0.0001
						Bonferroni	600 sec S vs TNF t(55) = 9.072, p<0.0001
						Bonferroni	800 sec S vs TNF t(55) = 9.418, p<0.0001
	**k**	Unpaired t-test	S WT = 6 TNFalpha WT = 10	t(14) = 2.193	0.0457	N/A	

**Figure**	**Panel**	**Test**	**Group size**	**Statistic**	**P value**	**Pair-wise comparisons**	**Statistic**
**Supplementary Fig. 1**	**a**	Unpaired t-test	Mnk1/2 +/+ = 9 Mnk1/2 -/- = 9	t(16) = 2.554	0.0212	N/A	
	**b**	Unpaired t-test	Eif4e +/+ = 6 Eif4e ki/ki = 7	t(11) = 2.984	0.0124	N/A	
	**c**	Repeated measures 2-way ANOVA (Time X Genotype)	Mnk1/2 +/+ = 13 Mnk1/2 -/- = 8	Main effect of genotype: F(1, 19) = 11.46	0.0031	N/A	

				Main effect of time: F(1, 19) = 56.28	< 0.0001	N/A	
				Time X Genotype Interaction: F(1, 19) = 7.120	0.0152	Bonferroni	1st exposure +/+ vs -/- t(38) = 4.300, p<0.001
	**d**	Repeated measures 2-way ANOVA (Time X Genotype)	Mnk1/2 +/+ = 13 Mnk1/2 -/- = 8	Main effect of genotype: F(1, 19) = 27.14	< 0.0001	N/A	
				Main effect of time: F(1, 19) = 0.7655	0.3925		
				Time X Genotype Interaction: F(1, 19) = 3.826	0.0653		
	**e**	Repeated measures 2-way ANOVA (Time X Genotype)	Mnk1/2 +/+ = 7 Mnk1/2 -/- = 14	Main effect of genotype: F(1, 19) = 6.430	0.0202	N/A	
				Main effect of time: F(1, 19) = 49.31	< 0.0001	N/A	
				Time X Genotype Interaction: F (1, 19) = 10.85	0.0038	Bonferroni	1st exposure +/+ vs -/- t=4.230 df=38, p<0.0001
	**f**	Repeated measures 2-way ANOVA (Time X Genotype)	Mnk1/2 +/+ = 7 Mnk1/2 -/- = 14	Main effect of genotype: F(1, 19) = 23.56	0.0001	N/A	
				Main effect of time: F(1, 19) = 7.729	0.0119	N/A	

				Time X Genotype Interaction: F(1, 19) = 2.698	0.1169	N/A
**Supplementary Fig. 2**	**a**	Repeated measures 2-way ANOVA (5-HT exposure X Genotype)	Eif4e +/+ = 7 cells<br>Eif4e ki/ki = 8 cells	Main effect of genotype: F(1, 13) = 1.253	0.6992	N/A
				Main effect of 5-HT exposure: F(1, 13) = 2.871	0.114	N/A
				Interaction Genotype X 5-HT exposure: F(1, 13) = 0.1561	0.2832	N/A
	**b**	Repeated measures 2-way ANOVA (5-HT exposure X Genotype)	Eif4e +/+ = 7 cells<br>Eif4e ki/ki = 8 cells	Main effect of genotype: F(1, 13) = 1.846	0.1974	N/A
				Main effect of 5-HT exposure: F(1, 13) = 3.200	0.097	N/A
				Interaction Genotype X 5-HT exposure: F(1, 13) = 0.006	0.9374	N/A
	**c**	Repeated measures 2-way ANOVA (5-HT exposure X	Eif4e +/+ = 7 cells<br>Eif4e ki/ki = 8 cells	Main effect of genotype: F(1, 13) = 2.347	0.1495	N/A

		Genotype)				
				Main effect of 5-HT exposure: F(1, 13) = 8.354	0.0126	N/A
				Interaction Genotype X 5-HT exposure: F(1, 13) = 3.068	0.1034	N/A
	**f**	Repeated measures 2-way ANOVA (5-HT exposure X Treatment)	S Eif4e ki/ki = 11 DN Eif4e ki/ki = 8	Main effect of treatment: F(1, 17) = 9.569	0.0066	N/A
				Main effect of 5-HT exposure: F(1, 17) = 2.301	0.1477	N/A
				5-HT exposure X Treatment Interaction: F(1, 17) = 0.094	0.7628	N/A
	**g**	Repeated measures 2-way ANOVA (5-HT exposure X Treatment)	S Eif4e +/+ = 10 DN Eif4e +/+ = 8 TNFa Eif4e +/+ = 9	Main effect of treatment: F(2, 24) = 0.5785	0.5684	N/A
				Main effect of 5-HT exposure: F(1, 24) = 0.0067	0.9356	N/A

				5-HT exposure X Treatment Interaction: F(2, 24) = 0.9776	0.3907	N/A	
**Supplementary Fig. 3**	**a**	Repeated measures 2-way ANOVA (Treatment X Genotype)	Eif4e +/+ = 5<br>Eif4e ki/ki = 6	Main effect of genotype: F(1, 9) = 15.38	0.0035	N/A	
				Main effects of treatment: F(1, 9) = 6.748	0.0288	N/A	
				Treatment X Genotype Interaction: F(1, 9) = 8.545e-005	0.9928	N/A	
	**b**	Repeated measures 2-way ANOVA (Treatment X Genotype)	Eif4e +/+ = 5<br>Eif4e ki/ki = 6	Main effect of genotype: F(1, 9) = 0.1409	0.7161	N/A	
				Main effect of treatment: F(2, 18) = 17.99	< 0.0001	Bonferroni	0.1 vs 0.3 dose: t(18) = 5.761, p<0.001
						Bonferroni	0.1 vs 0.2 dose: t(18) =3.31, p<0.05
				Treatment X Genotype Interaction: F (2, 18) = 4.778	0.0216	Bonferroni	No pairwise (+/+ vs ki/ki) comparisons significant
	**c**	Unpaired t-test with Welch's	Eif4e +/+ = 34<br>Eif4e ki/ki = 24	t(53.77) = 2.751	0.0081	N/A	

		correction					
	**d**	Unpaired t-test	Eif4e +/+ = 4<br>Eif4e ki/ki = 4	t(6) = 1.037	0.3398	N/A	
**Supplementary Fig. 4**	**b**	Repeated measures 2-way ANOVA (5-HT exposure X Genotype)	Eif4e +/+ = 8 cells<br>Eif4e ki/ki = 13 cells	Main effect of genotype: F(1, 19) = 14.55	0.0012		
				Main effect of 5-HT exposure: F(1, 19) = 14.99	0.001		
				5-HT exposure X Genotype Interaction: F(1, 19) = 18.45	0.0004	Bonferroni	Baseline +/+ vs ki/ki t(38)=0.094, p>0.05
						Bonferroni	5-HT +/+ vs ki/ki t(38)=5.714, p<0.0001
	**c**	Repeated measures 2-way ANOVA (5-HT exposure X Genotype)	Eif4e +/+ = 8 cells<br>Eif4e ki/ki = 13 cells	Main effect of genotype: F(1, 19) = 0.5311	0.475	N/A	
				Main effect of 5-HT exposure: F(1, 19) = 0.006023	0.939	N/A	
				Interaction Genotype X 5-HT exposure: F(1, 19) = 6.575	0.019	Bonferroni	No pairwise (+/+ vs ki/ki) comparisons significant

	**d**	Repeated measures 2-way ANOVA (5-HT exposure X Genotype)	Eif4e +/+ = 8 cells<br>Eif4e ki/ki = 13 cells	Main effect of genotype: F(1, 19) = 1.358	0.259	N/A
				Main effect of 5-HT exposure: F(1, 19) = 0.1745	0.6811	N/A
				5-HT exposure X Genotype Interaction: F(1, 19) = 0.0128	0.9113	N/A
	**e**	Repeated measures 2-way ANOVA (5-HT exposure X Genotype)	Eif4e +/+ = 8 cells<br>Eif4e ki/ki = 13 cells	Main effect of genotype: F(1, 19) = 0.3764	0.5472	N/A
				Main effect of 5-HT exposure: F(1, 19) = 0.1239	0.7289	N/A
				5-HT exposure X Genotype Interaction: F(1, 19) = 0.4198	0.5252	N/A
	**f**	2-way ANOVA (Treatment X Genotype)	S Eif4e +/+ = 6<br>F Eif4e +/+ = 6	Main effect of genotype: F(1, 26) = 7.514	0.0109	N/A
			S Eif4e ki/ki = 8<br>F Eif4e ki/ki = 10	Main effect of treatment: F(1, 26) = 23.34	<0.0001	N/A

				Treatment X Genotype Interaction: F(1, 26) = 0.261	0.6138	N/A
	g	2-way ANOVA (Treatment X Stress)	Control Sal = 5<br>Control Ket = 6	Main effect of stress: F(1, 19) = 0.5391	0.4718	N/A
			CSD Saline = 6<br>CSD Ket = 6	Main effect of treatment: F(1, 19) = 1.080	0.3117	N/A
				Treatment X Genotype Interaction: F(1, 19) = 0.011	0.9185	N/A
	h	2-way ANOVA (Treatment X Stress)	Control Sal = 5<br>Control Ket = 6	Main effect of stress: F(1, 19) = 0.3867	0.5515	N/A
			CSD Saline = 6<br>CSD Ket = 6	Main effect of treatment: F(1, 19) = 0.1324	0.72	N/A
				Treatment X Genotype Interaction: F(1, 19) = 0.3867	0.5414	N/A
**Supplementary Fig. 5**	a	2-way ANOVA (Gender X Genotype)	Males:<br>Eif4e +/+ = 6<br>Eif4e ki/ki = 7	Main effect of genotype: F(1, 29) = 0.2713	0.6064	N/A
			Females:<br>Eif4e +/+ = 10<br>Eif4e ki/ki = 10	Main effect of gender: F(1, 29) = 0.0335	0.8562	N/A

				Gender X Genotype Interaction: F(1, 29) = 3.812	0.0606	N/A
	**c**	2-way ANOVA (Gender X Genotype)	Males:<br>Eif4e +/+ = 9<br>Eif4e ki/ki = 7	Main effect of genotype: F(1, 33) = 0.3689	0.5478	N/A
			Females:<br>Eif4e +/+ = 10<br>Eif4e ki/ki = 11	Main effect of gender: F(1, 33) = 1.163	0.2886	N/A
				Gender X Genotype Interaction: F(1, 33) = 1.689	0.2028	N/A
	**e**	2-way ANOVA (Gender X Genotype)	Males:<br>Eif4e +/+ = 8<br>Eif4e ki/ki = 5	Main effect of genotype: F(1, 23) = 0.1774	0.6775	N/A
			Females:<br>Eif4e +/+ = 7<br>Eif4e ki/ki = 7	Main effect of gender: F(1, 23) = 0.4909	0.4906	N/A
				Gender X Genotype Interaction: F(1, 23) = 1.864	0.1854	N/A
**Supplementary Fig. 6**	**a**	Unpaired t-test (Mnk1/2 data)	Mnk1/2 +/+ = 9<br>Mnk1/2 -/- = 9	t(16) = 0.2797	0.7836	N/A
		Unpaired t-test (Eif4e data)	Eif4e +/+ = 11<br>Eif4e ki/ki = 10	t(19) = 1.529	0.1426	N/A
	**b**	Unpaired t-test (Mnk1/2 data)	Mnk1/2 +/+ = 7<br>Mnk1/2 -/- = 13	t(18) = 2.592	0.0184	N/A

		Unpaired t-test (Eif4e data)	Eif4e +/+ = 7<br>Eif4e ki/ki = 10	t(15) = 3.072	0.0078	N/A
	**c**	Unpaired t-test with Welch's correction	Mnk1/2 +/+ = 7<br>Mnk1/2 -/- = 8	t(7.134) = 2.384	0.048	N/A
	**d**	Unpaired t-test with Welch's correction	Mnk1/2 +/+ = 6<br>Mnk1/2 -/- = 12	t(14.61) = 2.225	0.0423	N/A
	**e**	Unpaired t-test	Eif4e +/+ = 5<br>Eif4e ki/ki = 13	t(16) = 1.313	0.2076	N/A
	**f**	Unpaired t-test with Welch's correction	Eif4e +/+ = 7<br>Eif4e ki/ki = 9	t(9.297) = 2.283	0.0475	N/A
	**g**	Unpaired t-test with Welch's correction	Eif4e +/+ = 5<br>Eif4e ki/ki = 8	t(7.679) = 2.877	0.0215	N/A
	**h**	2-way ANOVA (Treatment X Genotype)	S Eif4e +/+ = 4<br>F Eif4e +/+ = 4	Main effect of genotype: F(1, 12) = 5.118	0.043	N/A
			S Eif4e ki/ki = 4<br>F Eif4e ki/ki = 4	Main effect of treatment: F(1, 12) = 14.86	0.0023	N/A
				Treatment X Genotype Interaction: F(1, 12) = 0.02538	0.8761	N/A

**Supplementary Fig. 7**	**a**	2-way ANOVA (Treatment X Genotype)	S Mnk1/2 +/+ = 9 DN Mnk1/2 +/+ = 10	Main effect of genotype: F(1, 29) = 33.39	< 0.0001	N/A	
			S Mnk1/2 -/- = 6 DN Mnk1/2 -/- = 8	Main effect of treatment: F(1, 29) = 9.189	0.0051	N/A	
				Treatment X Genotype Interaction: F(1, 29) = 5.931	0.0213	Bonferroni	S Mnk1/2 +/+ vs S Mnk1/2 -/- t(29) = 5.525, p<0.001
						Bonferroni	S Mnk1/2 -/- vs DN Mnk1/2 -/- t(29) = 3.589, p<0.01
	**b**	2-way ANOVA (Treatment X Genotype)	S Mnk1/2 +/+ = 8 DN Mnk1/2 +/+ = 10	Main effect of genotype: F(1, 32) = 6.686	0.0145	N/A	
			S Mnk1/2 -/- = 8 DN Mnk1/2 -/- = 10	Main effect of treatment: F(1, 32) = 5.572	0.0245	N/A	
				Treatment X Genotype Interaction: F(1, 32) = 7.141	0.0118	Bonferroni	S Mnk1/2 +/+ vs S Mnk1/2 -/- t(32) = 3.527, p<0.001
						Bonferroni	S Mnk1/2 -/- vs DN Mnk1/2 -/- t(32) = 3.559, p<0.01

Supplementary Figure 9

Uncropped blot images

From Figure 3e

Phosphorylated eIF4E:

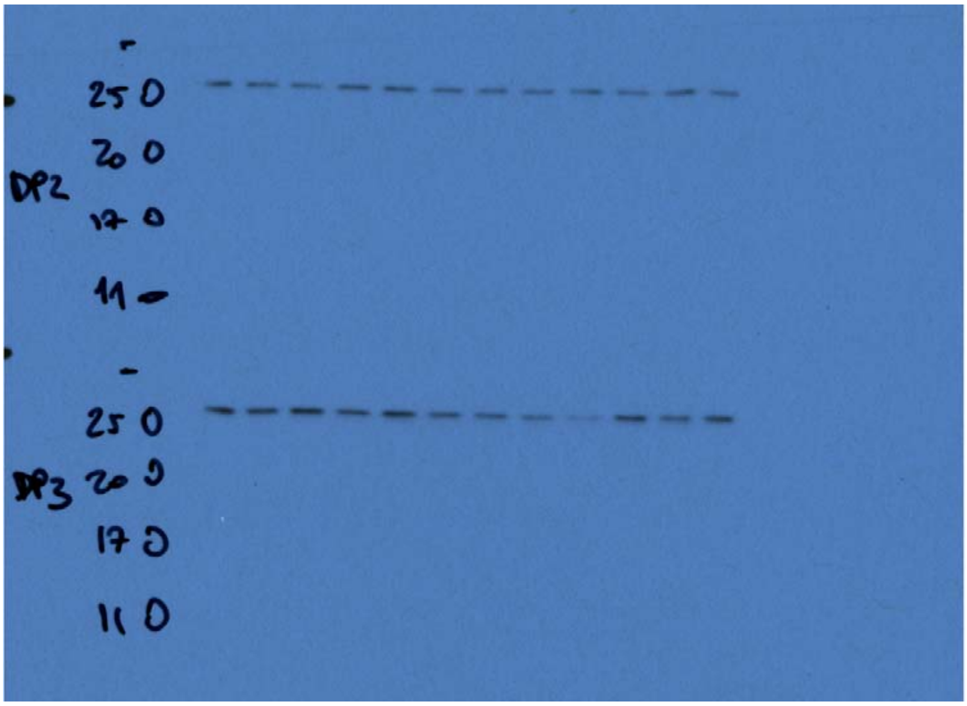

Total eIF4e:

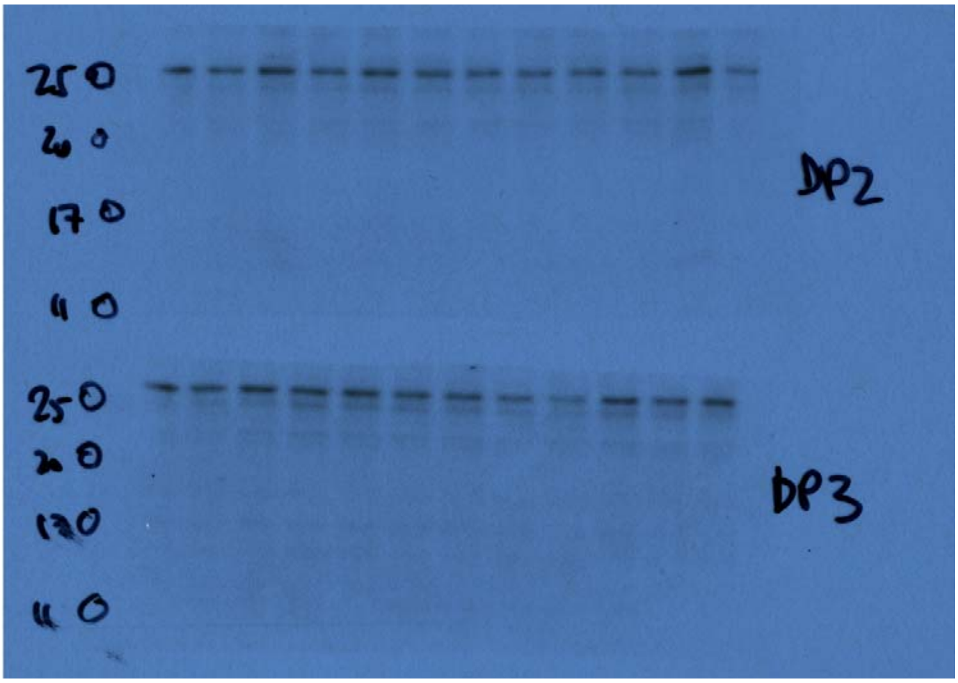

Supplementary Figure 9 (continued)

GAPDH:

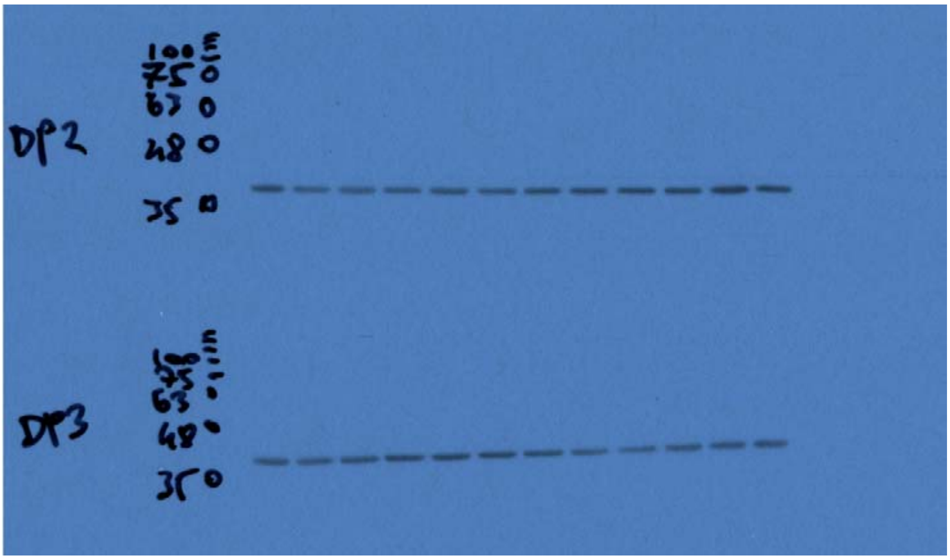

From Figure 4b:

Phosphorylated eIF4E:

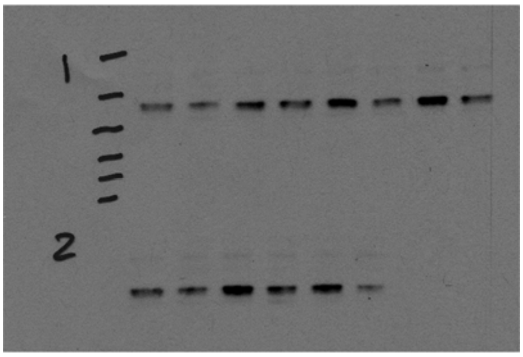

Total eIF4E:

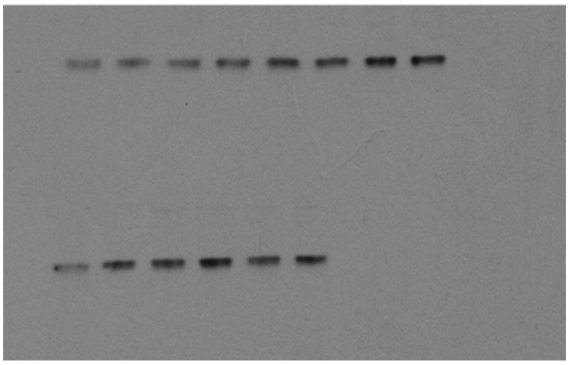

Supplementary Figure 9 (continued)

GAPDH:

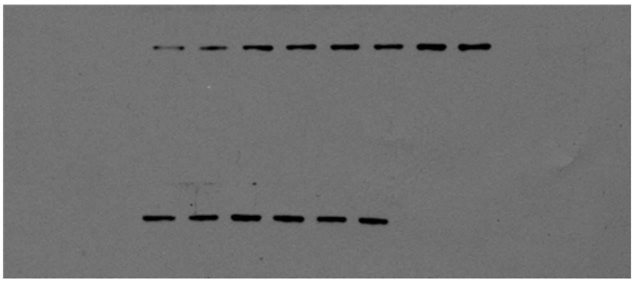

From Figure 5e-f:

IκBα and phosphorylated eIF4E:

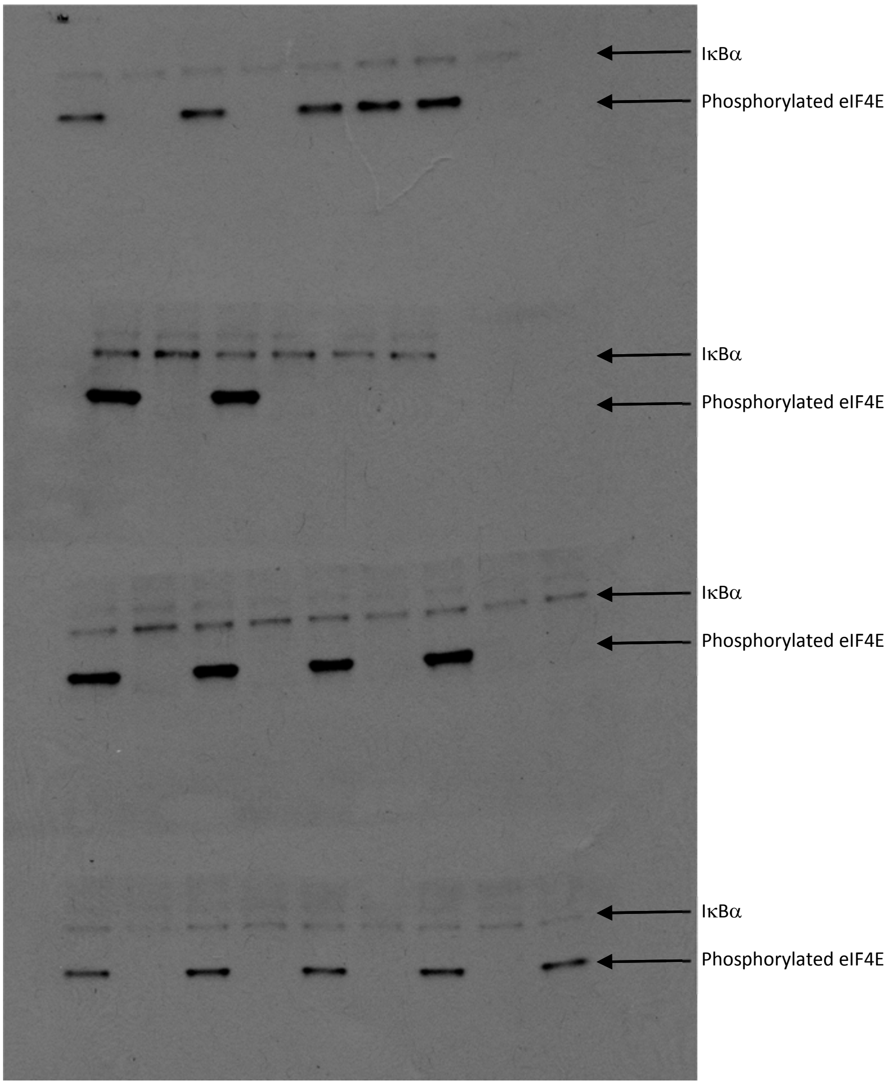

Supplementary Figure 9 (continued)

Total eIF4E:

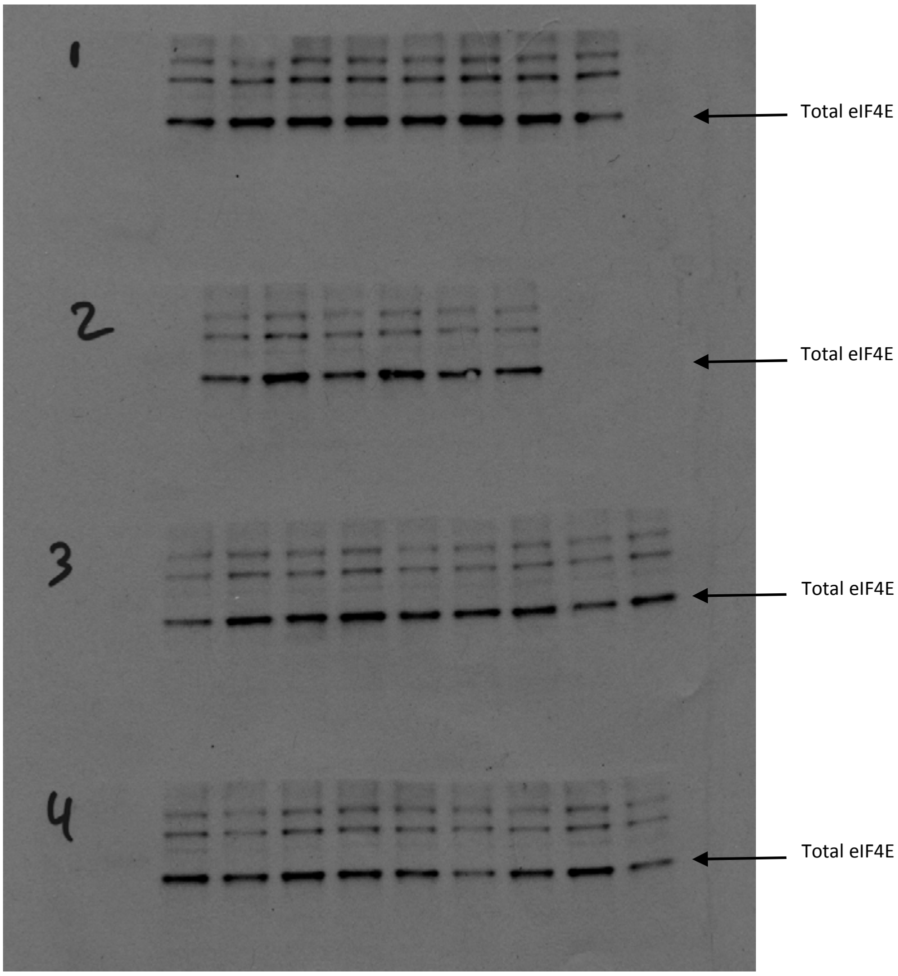

Supplementary Figure 9 (continued)

GAPDH:

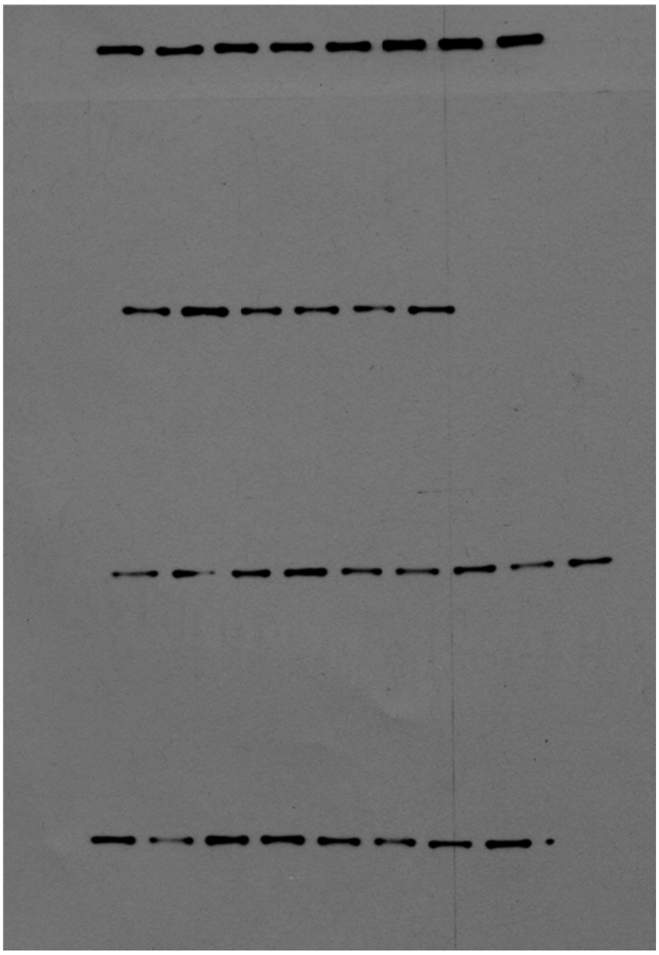

Supplementary Figure 9 (continued)

From Supplementary Figure 4i:

Phosphorylated eIF4E:

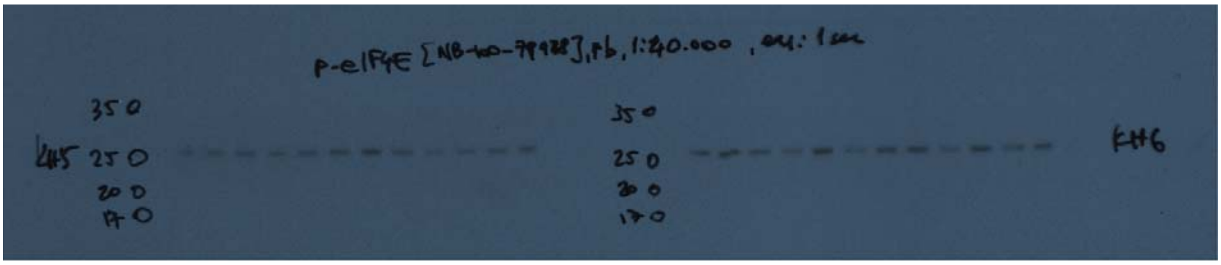

Total eIF4e:

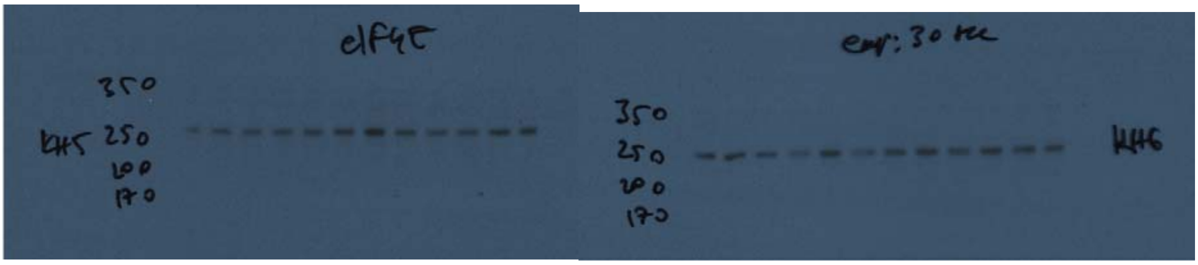

β-actin:

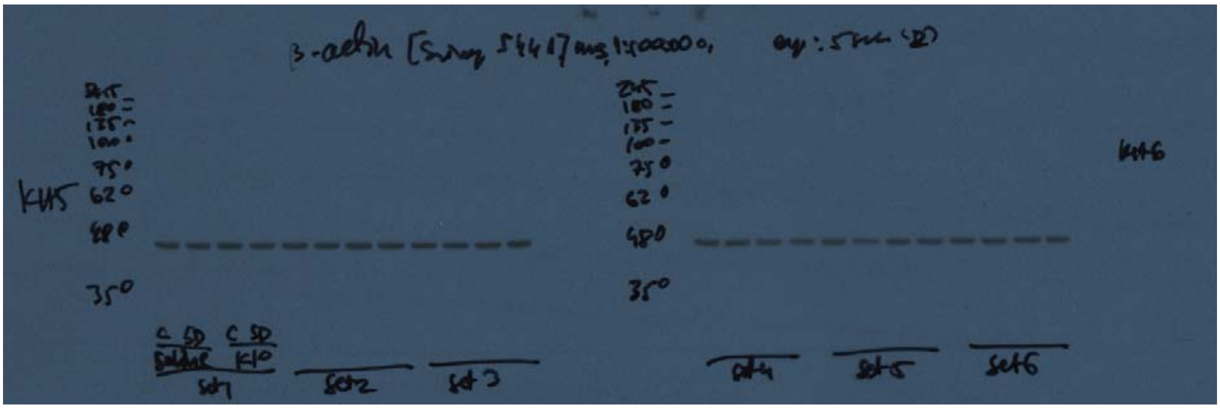

Supplement: Supplementary file 1 — Supplementary Information [file 41467_2018_4883_MOESM1_ESM.pdf]
