# Supplementary material for: Translational control of depression-like behavior via phosphorylation of eukaryotic translation initiation factor 4E
Source: Nat Commun. 2018 Jun 25;9:2459. doi: 10.1038/s41467-018-04883-5 (PMC6018502; doi:10.1038/s41467-018-04883-5)
Supplement: Supplementary file 2 — Peer Review File [file 41467_2018_4883_MOESM2_ESM.pdf]

Editorial Note: This manuscript has been previously reviewed at another journal that is not operating a transparent peer review scheme. This document only contains reviewer comments and rebuttal letters for versions considered at Nature Communications.

REVIEWERS' COMMENTS:

Reviewer #1 (Remarks to the Author):

The authors have addressed all of my comments. This is a very nice study. One minor quibble. Throughout the text the authors now use the term "depression-like behaviors" which is appropriate. Likewise, the title should be changed to "depression-like behaviors" instead of "depression".

Reviewer #2 (Remarks to the Author):

I am impressed with the additional amount of work performed by the authors. I have no additional comments.

Reponse to reviewers' comments:

Reviewer #1 (Remarks to the Author): The authors have addressed all of my comments. This is a very nice study. One minor quibble. Throughout the text the authors now use the term "depression-like behaviors" which is appropriate. Likewise, the title should be changed to "depression-like behaviors" instead of "depression".

We thank the reviewer for the encouraging comments. We have modified the title to depression-like behaviours.

Reviewer #2 (Remarks to the Author): I am impressed with the additional amount of work performed by the authors. I have no additional comments.

We also thank this reviewer for the her/his positive comment.
